# Self-Assembly of 3D-Printed Multiscale Micropillar-Based Organic Electrochemical Transistors for Ultrasensitive Dopamine Sensing

**DOI:** 10.1021/acsnano.5c02784

**Published:** 2025-08-05

**Authors:** Xinzhao Zhou, Liwen Zhang, Shengbin Zhang, Jing Liang, Ke Zhang, Zehui Zhao, Song Zhao, Yan Wang, Yurun Guo, Deyuan Zhang, Lei Jiang, Huawei Chen

**Affiliations:** † School of Mechanical Engineering and Automation, 12633 Beihang University, Beijing 100191, China; ‡ College of Mechanical and Transportation Engineering, China University of Petroleum, Beijing 102249, China; § Laboratory of Bioinspired Smart Interface Science, Technical Institute of Physics and Chemistry, 12381 Chinese Academy of Sciences, Beijing 100190, China; ∥ Beijing Advanced Innovation Centre for Biomedical Engineering, Beihang University, Beijing 100191, China

**Keywords:** ultrasensitive electrochemical sensing, aerosol jet printing, organic electrochemical transistor, dopamine sensor, multiscale 3D structure

## Abstract

Organic electrochemical transistors (OECTs) with self-amplification have emerged as a promising approach for dopamine monitoring. However, the planar gate electrode structures in current OECTs significantly hinder the improvement of sensitive dopamine detection. Here, we develop an aerosol jet three-dimensional (3D) printing method to generate multiscale micropillar-based OECTs with tunable nanofeatures for ultrasensitive DA detection. Temperature-induced self-assembly nanoclusters are explored to replace atomized microdroplets to generate multiscale micropillars, and their diameter-tuning mechanism is clarified to enable on-demand nanofeature control. We fabricate micropillar electrodes with varying surface morphologies, array numbers, and heights, and uncover their micronano synergic enhancement effects on the mass transfer, catalytic efficiency, and ion migration that determine the sensing performance of the OECT. When the nanocluster diameter is 540 nm, the OECT achieves a maximized sensitivity of 254 mV/decade and an ultralow dopamine detection limit of 0.6 fM, which is 6 orders of magnitude lower than current methods. Integrated with a flexible circuit, the device allows real-time, wireless, and highly sensitive dopamine sensing in the rabbit brain, demonstrating its potential for future applications in the precise diagnosis and treatment of mental illnesses.

## Graphical abstract

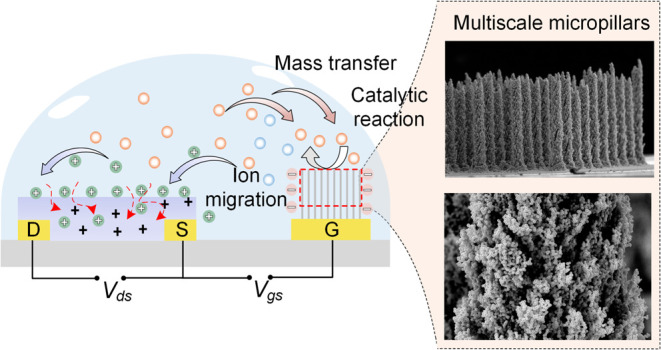

Mental illnesses such as Parkinson’s disease, major depression, and schizophrenia have increasingly afflicted over 300 million individuals and caused more than one million deaths in recent years.^1–4^ Dysregulated dopamine (DA) signaling in the central nervous system is a typical feature of these illnesses (Figure 1A, left).^5–7^ Therefore, accurate monitoring of DA is urgent for understanding its pathogenesis, early diagnosis, and precise treatment. Nevertheless, the extremely low concentration of DA in the human body (pM-nM levels) and the presence of various interfering substances pose significant challenges to achieving highly sensitive DA detection.^7–9^

**Figure 1:**
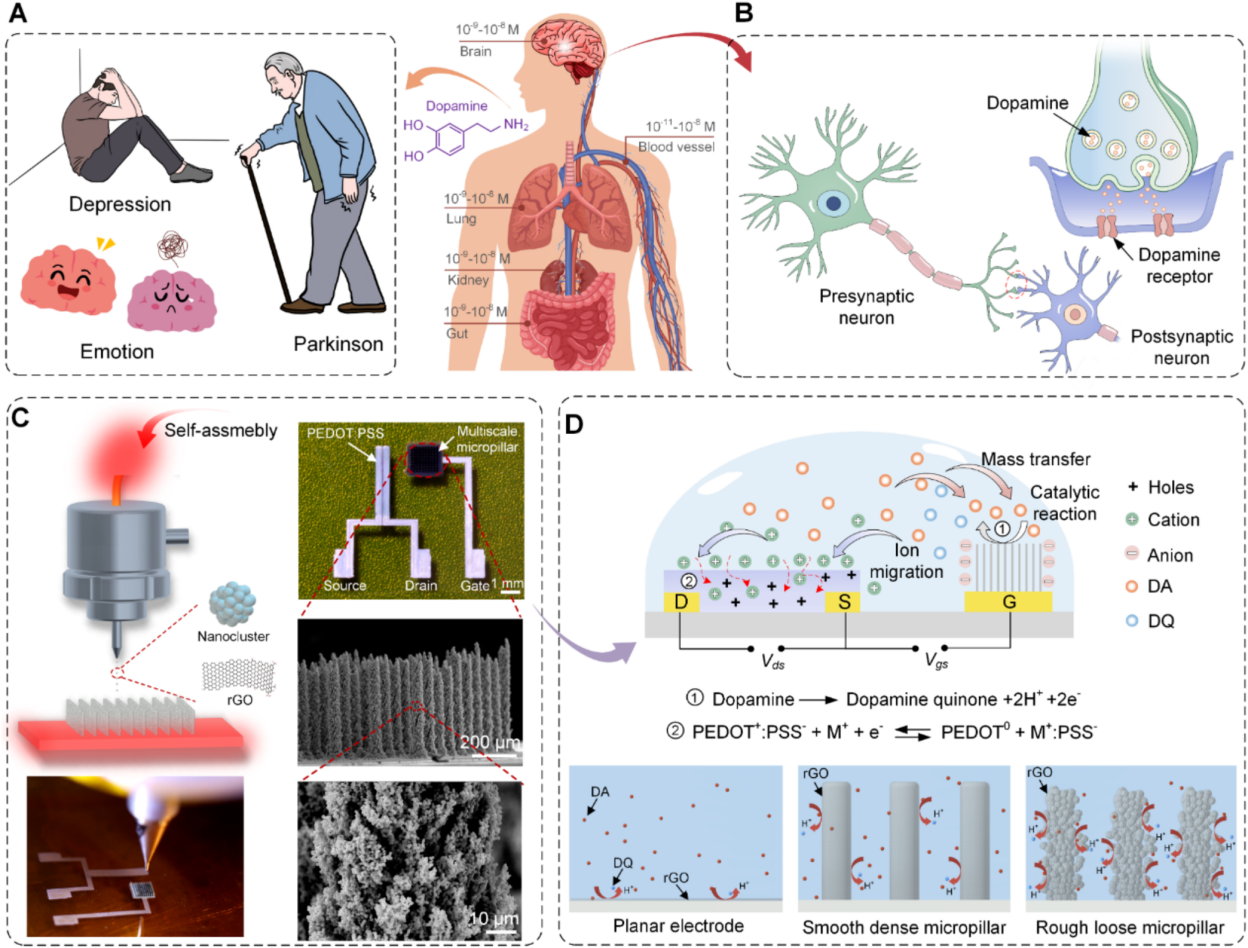
Ultrasensitive DA OECT based on a nanocluster-assembled multiscale micropillar gate electrode. (A) DA concentration distribution in the human body and neurological diseases resulting from abnormal DA secretion. (B) Schematic diagram of DA transmission in biological neurons. DA is released from the presynaptic neuron and bonded to specific receptors on the postsynaptic neuron, triggering the excitation of the next neuron. (C) AJP with temperature-induced self-assembly and DA OECT with a nanocluster-assembled multiscale micropillar gate electrode. (D) Top: schematic illustration of the OECT working mechanism for DA detection. DA is oxidized to dopamine quinone (DQ) at the gate electrode, altering the gate potential and triggering additional ion injection into the semiconductor channel to change the source-drain current. Bottom: Compared to the planar and smooth dense micropillar electrodes, more DA accumulates and reacts on the multiscale micropillar electrode.

OECTs have been widely adopted for DA detection owing to their excellent signal amplification, superior signal-to-noise ratio, and biocompatibility.^10–14^ In OECTs, the gate electrode enables the DA electrochemical reaction and ultimately determines the sensing behavior, functioning similarly to a DA receptor in the nervous system (Figure 1B). Hence, to maximize the DA reaction and improve the sensitivity, it is critical to enhance the gate electrode’s reaction area and DA catalytic efficiency. Although nanofunctional materials such as reduced graphene oxide (rGO) and platinum nanoparticles have been modified on gate electrodes to address these needs,^15–19^ most of these gate electrodes remain limited to planar structures. Their low catalyst loading, limited reaction areas, and linear diffusion result in the detection limits remaining at the nM level. To further improve the DA detection limit from nM to pM or even fM, high-surface-area multiscale three-dimensional (3D) electrodes provide an effective strategy by enhancing diffusion and promoting analyte accumulation.^20–22^ However, the application and enhancement mechanisms of multiscale gate electrodes in the OECTs remain largely unexplored. Furthermore, precise control over the micro- and nanoscale features of multiscale electrodes is essential for realizing their full potential. Hence, a highly accurate, controllable, and reproducible fabrication method is urgently required.

Here, we develop an aerosol jet 3D printing (AJP) method to generate multiscale micropillar-based OECTs with tunable nanofeatures (Figure 1C). Temperature-induced self-assembly nanoclusters are used to replace the atomized microdroplets to construct multiscale micropillars. We vary the surface morphologies, array numbers, and heights of the micropillars to tune their multiscale features and compare their electrochemical and DA detection performances. We demonstrate that the micronano synergy of the multiscale micropillar significantly enhances the DA mass transfer, catalytic reaction efficiency, and ion migration in an OECT (Figure 1D), termed the triple-enhancement effect. Such a triple-enhancement effect not only improves the DA detection limit by 6 orders of magnitude, from nM to fM levels, but also enhances sensitivity and selectivity. By integrating with a flexible printed circuit board (FPCB) to create an implant device, its ability for real-time and highly sensitive DA monitoring is validated by implanting it in the rabbit brain, demonstrating its significant potential for the diagnosis and treatment of related mental illnesses.

## Results

### Temperature-Induced Self-Assembly 3D Printing for Multiscale Structures

AJP uses ultrasonic energy to disperse nanoparticle (NP) ink into microsized droplets (1–5 μm) and employs precise sheath gas focusing to achieve high-resolution, multimaterial, and 3D structure fabrication.^23–27^ However, due to the lack of nanoscale regulation methods, the current microdroplet-based AJP faces challenges in directly fabricating micronano multiscale structures, ranging from tens of nanometers to hundreds of micrometers.^21,23,28–30^ To achieve a multiscale gate electrode with a high surface area, a two-stage heating method, including preheating and substrate heating, has been introduced into the AJP process (Figures 2A and S1). The initial preheating step is applied at the transferring tube between the ultrasonic atomizer and the deposition head at a temperature of *T*_pre_ (Figure 2A). Due to the heat-induced evaporation of the solvent in the microdroplet at the transition temperature (*T*_tra_), the internal Ag NPs (30–50 nm) aggregate and self-assemble into perfect spherical nanoclusters (NCs), driven by the microdroplets’ capillarity forces (Figure 2A­(i),B). Such a self-assembly process occurs only when *T*_pre_ exceeds *T*_tra_ (Figures 2B and S2), which is closely related to the carrier gas flow rate, increasing from 35 to 50 °C as the flow rate rises from 20 to 30 sccm (Figure S3). Remarkably, these nanoclusters can still maintain their independence and do not merge, even after they undergo collisions during transport and deposition (Figure S4).

**Figure 2:**
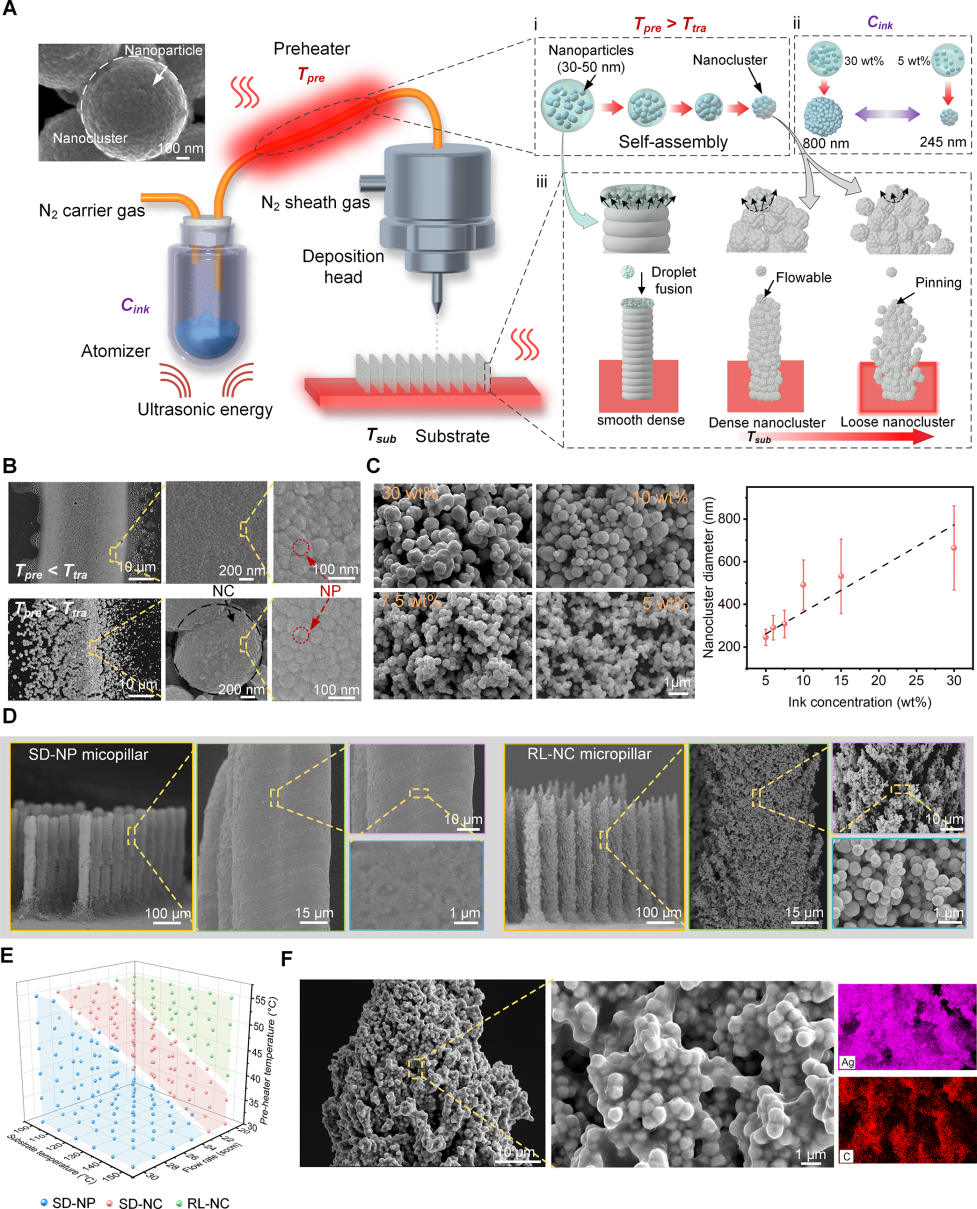
Temperature-induced self-assembly aerosol jet 3D printing for multiscale structures. (A) Schematic of the AJP system consisting of an ultrasonic atomizer, preheater, movable deposition head, and substrate. The micronano multiscale structure is adjusted by tuning the preheating temperature *T*_pre_ (i), the Ag NP ink concentration *C*_ink_ (ii), and the substrate heating temperature *T*_sub_ (iii). (B) SEM images of tracks printed at a speed of 1 mm/s at different *T*_pre_ values. (C) SEM images of the NCs at different *C*_ink_, and the relationship between *C*_ink_ and the NC diameter. (D) SEM images of SD-NP and RL-NC micropillar arrays (450 μm height, 60 μm diameter, and 200 μm center distance). Images obtained at higher magnifications show the characteristics of the NPs and NCs. (E) Printing conditions for fabricating different types of micropillar structures. The formation of SD-NP, SD-NC, and RL-NC micropillars is achieved by adjusting *T*_pre_, carrier gas flow rate, and *T*_sub_. (F) SEM images and energy-dispersive X-ray spectroscopy (EDX) of rGO and Nafion normally wrapped on the surface of the NCs.

Since the diameter of the NC is determined by the number of NPs in each microdroplet during self-assembly, it can be precisely adjusted over a wide range by controlling the concentration of the Ag NP ink (*C*_ink_) (Figure 2A­(ii)). By gradually diluting *C*_ink_ from 30 to 5 wt % with deionized water, the NC diameter was reduced from ∼800 to ∼200 nm with high uniformity (Figures 2C and S5). After passing through the preheater, two conditions occur under different *T*_pre_ values: microdroplets containing NPs (*T*_pre_ < *T*_tra_) and NCs with partial residual solvent (*T*_pre_ > *T*_tra_), which are separately deposited onto the substrate. Under second-stage heating with a substrate temperature of *T*_sub_, the surface tension of the residual solvent allows the NPs and NCs to adhere and vertically stack into a 3D structure on the substrate. Moreover, the evaporation speed of the residual solvent could be further adjusted by changing *T*_sub_. With lower *T*_sub_, the residual solvent leads to high fluidity of NP microdroplets and NCs (Figure 2A) (iii), which are eventually compressed into a dense structure by gravity and nanocapillarity force (Figures 2D, left and S6). For NCs deposited at higher *T*_sub_, the rapid evaporation of the residual solvent significantly reduces the NC fluidity, causing them to be pinned and solidified instantaneously (Figure 2A iii and Movie S1), which leads to the formation of loose NC multiscale structures with a much higher surface area (Figure 2D, right). Such loose NC multiscale structures exhibit a high degree of uniformity across the entire micropillar (Figure S7), in contrast to the microbranches formed solely at the base through the aerosol turbulence method.^28^

Utilizing this temperature-induced self-assembly AJP, a smooth dense Ag NP/NC (SD-NP/SD-NC) micropillar array and a rough loose Ag NC (RL-NC) micropillar array were precisely designed and fabricated (Figures 2D and S6), with their ideal printing conditions determined (Figures 2E and S8). Full sintering at 300 °C for 30 min effectively fused these NPs and NCs, ensuring high conductivity even in the RL-NC micropillar, which also exhibits excellent structural stability under a 2 m/s water flow impact (Figures S9 and 10). The SD-NP micropillar, SD-NC micropillar, and RL-NC micropillar assembled from different NC diameters were printed as the electrodes in this study, featuring an outer diameter (*D*_out_) of 60 μm, an inner diameter (*D*_in_) of 20 μm, a height ranging from 150 to 450 μm, and a center distance (*C*_d_) of 200 μm (Figure S11). Among them, the RL-NC micropillar assembled from 550 nm NC exhibits the highest electroactive surface area (EASA), showing an increase of 441 and 50% compared to the planar electrode and the SD-NP micropillar electrode, respectively (Figure S12).

Moreover, to improve the DA catalytic performance^31–33^ and enhance the specific detection ability of the electrode,^15,34^ rGO ink containing 5 wt % Nafion was printed using the multimaterial AJP to uniformly cover the micropillars. Benefiting from π–π and electrostatic interactions,^35^ rGO nanosheets are uniformly wrapped around the micropillars. The RL-NC micropillar allows for higher rGO loading compared to the SD-NP micropillar due to its excellent surface area, leading to its higher catalytic capacity for DA (Figures 2F, and S13, and 14). In addition, the rGO and Nafion coatings maintain the surface morphology of the RL-NC micropillar (Figures 2F and S15) and significantly enhance the surface wettability, which is beneficial for DA detection (Figure S16).

### Electrochemical Characterizations of the Micropillar Electrode in a Three-Electrode System and MSDA-OECT

To assess the electrochemical performance, the different types of micropillar electrodes mentioned above were used as the working electrodes and tested in a three-electrode electrochemical sensing system (Figure 3A). Finite element simulation results indicate that the micropillar electrode exhibits uniform linear and radial diffusion due to its 3D geometry, in contrast to planar electrodes that display only linear diffusion. This enhanced diffusion leads to a thinner diffusion layer and a higher concentration gradient, which could enhance the redox current (Figures 3B and S17).^21,36,37^ Notably, nanoclusters with nanocurvature on the surface of the RL-NC micropillar can significantly enhance the electric field to improve electrocatalytic activity and reduce concentration polarization^38,39^ (Figures 3C and S18), generating multiple high-flow regions that further enhance analyte diffusion (Figure 3B right). Finite element simulations reveal that the maximum electric field intensity at the nanocluster tip is 3.2 times greater than that of the SD-NP micropillar (Figure S18). However, due to the electrostatic shielding effect within the array electrode, this nanocluster-induced electric field enhancement is confined to the tips of the inner RL-NC micropillars and the entire outer surface of the outermost micropillars, exhibiting strong spatial variations (Figure S19). By cyclic voltammetry (CV) testing in the acetate buffer solution with 5 mM [Ru (NH)_6_]^3+^, the peak current of the RL-NC micropillar array electrodes assembled from 530 nm NC is ∼9.8 and ∼1.7 times higher than that of planar and SD-NP micropillar array electrodes (Figure 3D), respectively, which demonstrates a strong diffusion enhancement from the multiscale micropillar structure.

**Figure 3:**
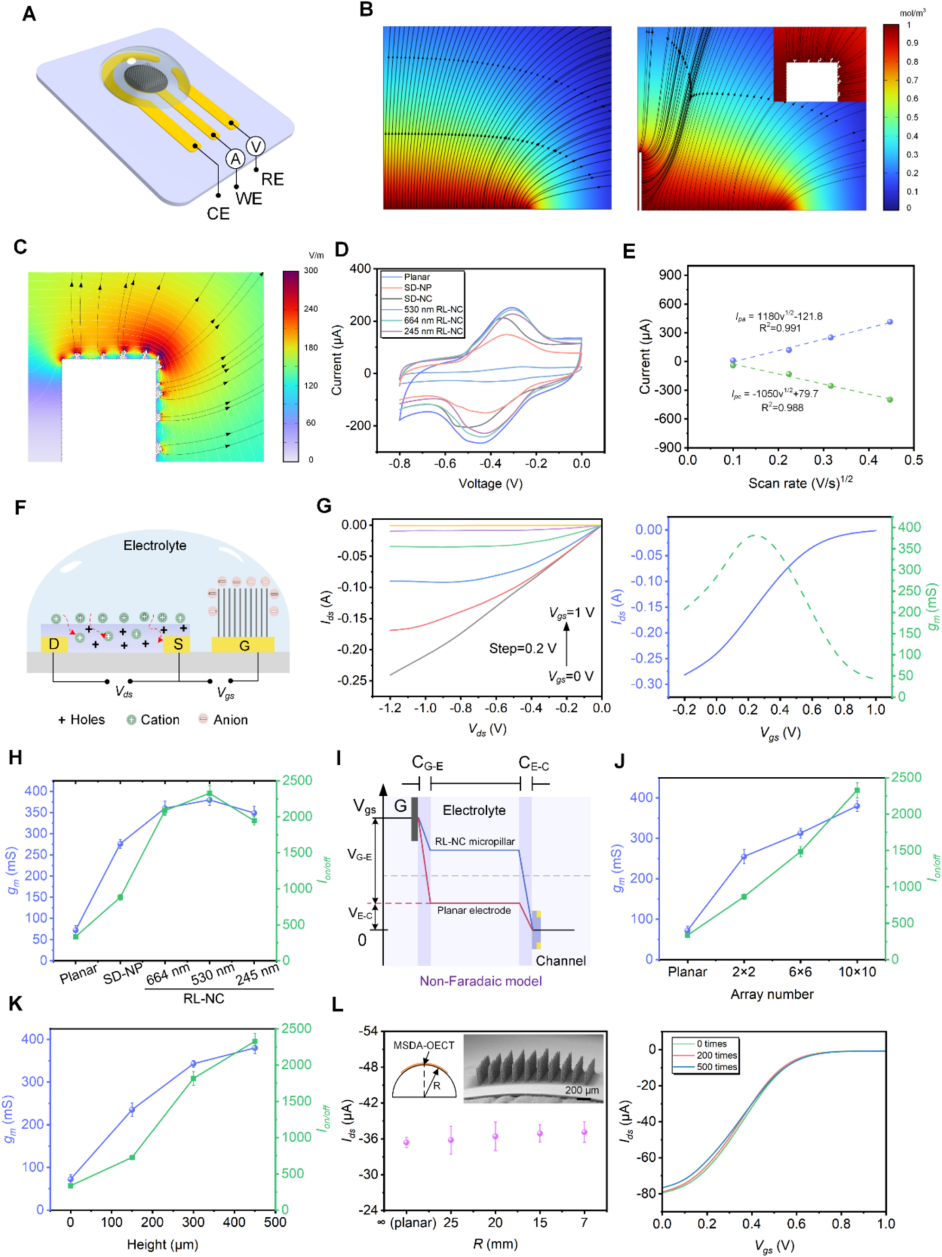
Electrochemical characterization of the multiscale micropillar electrode and MSDA-OECT. (A) Schematic of a three-electrode electrochemical sensing system, including the counter, working, and reference electrodes. (B) COMOSOL simulation of diffusion streamlines for the planar electrode and half of the single RL-NC micropillar electrode. The inset shows a magnified view of the diffusion streamlines around the top nanocluster region. (C) Magnified view of the electric field distribution at the tip of the nanocluster on the RL-NC micropillar (half of a single micropillar and 530 nm NC). (D) CV comparison of planar electrode, SD-NP/NC micropillar, and RL-NC micropillar array electrodes. The CV tests were conducted in an acetate buffer solution (100 mM, pH 4.5) with 5 mM [Ru (NH)_6_]^3+^ mediator at a scan rate of 100 mV/s. (E) Anodic and cathodic peak currents of the RL-NC micropillar array electrode vs square root of scan rate (0.01–0.2 V/s). (F) Schematic of the MSDA-OECT operating in the non-Faradaic mode. By applying *V*_gs_, anions and cations accumulate at the gate/electrolyte interface and electrolyte/channel interface, respectively, forming two double-layer capacitors (*C*_G‑E_, C_E‑C_). (G) Output and transfer characteristic curves of the MSDA-OECT, tested in PBS buffer solution (50 mM, pH 7.4) using an RL-NC micropillar array with a height of 450 μm and 10 × 10 configuration. (H) Effects of gate electrode type on the OECT *g*_m_ and on/off ratio. Error bars represent the standard error of the mean (*n* = 3). (I) Equivalent circuit and potential diagram of the OECT operating in a non-Faradaic model. The potential drops at both the gate/electrolyte and electrolyte/channel interfaces, assuming that the potential change in the electrolyte is negligible. (J, K) Effects of the micropillar array number and height on g_m_ and on–off ratio. Error bars represent the standard error of the mean (*n* = 3). (L) Current response of the MSDA-OECT at different bending radii (source-drain voltage (*V*_*ds*_) = −0.4 V, *V*_gs_ = 0.2 V), and transfer characteristic curves after bending 200 and 500 times at a radius of 7 mm. Error bars represent the standard error of the mean (*n* = 3). The inset shows the schematic diagram of the bending axis (*R*) and the SEM image of the RL-NC micropillar array with a bending radius of 7 mm.

To further evaluate the effect of the multiscale micropillar electrodes on the mass transfer of the analyte, a scan rate study was performed. The results show that the anodic and cathodic peak currents (*I*_pa_, *I*_pc_) of the RL-NC micropillar array electrode vary linearly with the square root of the scan rate, validating the typical diffusion-controlled process occurring on the electrode surface (Figure 3E and S20). According to the Rendles–Sevick equation,^40^ the RL-NC micropillar array electrode exhibits an effective diffusion coefficient (*D*_0_) of ∼3.29 × 10^–5^ cm^2^/s, which is 14.4 times higher than that of the planar electrode (Table S1). The effects of multiscale RL-NC micropillar electrodes with varying heights and array numbers on mass transfer were further characterized. The redox current increases with micropillar height but plateaus at 10 × 10 array with a fixed electrode size (2 × 2 mm^2^), likely due to the overlapping diffusion regions that reduce mass transfer efficiency (Figure S21).^41^

The excellent mass transfer capacity of the multiscale RL-NC micropillar makes it an ideal candidate for use as the gate electrode in the DA OECT. The multiscale DA OECT (MSDA-OECT) was fully printed by AJP (Figures S22, 23 and Movie S2) and characterized in phosphate buffer solution (PBS, 0.05 mM, pH 7.4) for semiconductor analysis (Figure 3F). Representative output and transfer characteristics, along with the transient current response, demonstrate that the MSDA-OECT has typical gate modulation behavior and fast response performance (Figures 3G and S24). Notably, the reaction between the Cl^–^ in PBS and Ag generates a Faradaic current that could potentially impact the gate-source current (*I*_gs_) and interfere with the performance of OECTs.^42^ Fortunately, the uniform wrapping of rGO and Nafion on the micropillar electrode isolates this reaction, eliminating the Faradaic current and ensuring a stable, continuous measurement (Figure S25). By comparing the OECTs with varied gate electrodes, MSDA-OECT with RL-NC micropillar assembled from 530 nm NC exhibits the best performance with an on/off ratio of 2.3 × 10^3^ and transconductance (*g*_*m*_) of 380 mS, which is 5.3 and 1.4 times higher than the *g*_m_ of OECT with planar and SD-NP micropillar gate electrodes, respectively (Figure 3G,H). Such performance enhancement is attributed to the excellent surface area of the RL-NC micropillar gate electrode, which increases the gate/electrolyte capacitance (*C*_G‑E_) and leads to a greater voltage drop at the electrolyte/channel interface, according to Bernard’s model^43^ (Figure 3I). Electrochemical impedance spectroscopy (EIS) of the RL-NC micropillar gate electrode and semiconductor channel reveals that the *C*_G‑E_ reaches 77 μF, while the electrolyte/channel capacitance (*C*_E‑C_) is 3.1 μF (Figure S26). The resulting *C*_G‑E_/*C*_E‑C_ ratio, substantially exceeding 10, ensures effective gating and facilitates more efficient ion migration and cation injection into the channel, enhancing the amplification capacity of the OECT.^44,45^ As the height and array number increase, *g*_*m*_ and the on/off ratio of the MSDA-OECT increase linearly (Figure 3J,K). Furthermore, the exceptional mechanical stability and flexibility of the RL-NC micropillar array enable the MSDA-OECT to maintain a stable and consistent sensing capability under various bending states, even after 500 bending cycles (Figure 3L). Considering the impact of the electrode type on electrochemical performance, OECT amplification, and device miniaturization, the optimized RL-NC micropillar gate electrode (assembled from 530 nm NC, 450 μm height, and 10 × 10 array) is selected for highly sensitive DA detection.

### In Vitro DA Detection Using a MSDA-OECT

An MSDA-OECT platform integrated with a microfluidic channel was used for in vitro DA detection (Figure 4A). Unlike the MSDA-OECT operating in non-Faradaic mode in pure PBS solution, when DA is detected, a Faradaic current is generated due to the oxidation of DA at the gate electrode. This alters the double-layer capacitance and increases the electrolyte/semiconductor interface potential, which pumps additional cations into the semiconductor channel to change the source-drain current (*I*_ds_) (Figures 1D and 4B). Such interface potential changes can be regarded as an increase in the effective gate voltage (*V*_geff_) applied on the OECT, which is related to the concentration of DA and defined as^46,47^

1 $$V_{\mathrm{geff}}=V_{\mathrm{gs}}+\left(1+\gamma \right)\frac{\mathrm{kT}}{\mathrm{ne}}\log\left[C_{\mathrm{DA}}\right]+\mathrm{constant}$$

where γ is the ratio between *C*_E‑C_ and *C*_G‑E_, *k* is the Boltzmann constant, *T* is the absolute temperature, *n* is the number of electrons transferred during the oxidation of DA (*n* = 2), *e* is the elementary charge of an electron, and *C*_DA_ is the DA concentration. Given the significant influence of *V*_gs_ on the double-layer capacitance and DA oxidation behavior, the normalized response (NR) of *I*_ds_ at different *V*_gs_ values was investigated to determine the optimal *V*_gs_ for DA detection. Specifically, the NR at a certain concentration is calculated by the following formula

2 $$\mathrm{NR}=\left|\left(I_{\mathrm{ds}}^{C_{\mathrm{DA}}}-I_{\mathrm{ds}}^{0}\right)/I_{\mathrm{ds}}^{0})\right|$$

where *I*_ds_^*C*_DA_^ is the *I*_ds_ with a certain concentration of DA, and *I*_ds_^0^ is the *I*_ds_ without the addition of DA. The NR changes obviously within the *V*_gs_ range of 0.2–0.5 V in the DA environment, with the maximum NR occurring at *V*_gs_ = 0.4 V (Figure 4C). Moreover, since the interfering substances uric acid (UA), ascorbic acid (AA), and serotonin (5-HT) coexist with DA in body fluids and have similar oxidation potentials, an optimized *V*_gs_ is crucial to enhance the selective detection of DA (Figure S27). By comparing the NR changes of UA, AA, and 5-HT, the operating voltage for *V*_*gs*_ was set to 0.2 V. Once the optimal *V*_gs_ for DA detection was determined, to achieve the highest amplification efficiency and modulation capability at *V*_gs_ = 0.2 V, the geometry of the semiconductor channel layer was further optimized with a width-to-length ratio (*W/L*) of 50 (Figure 4D).

**Figure 4:**
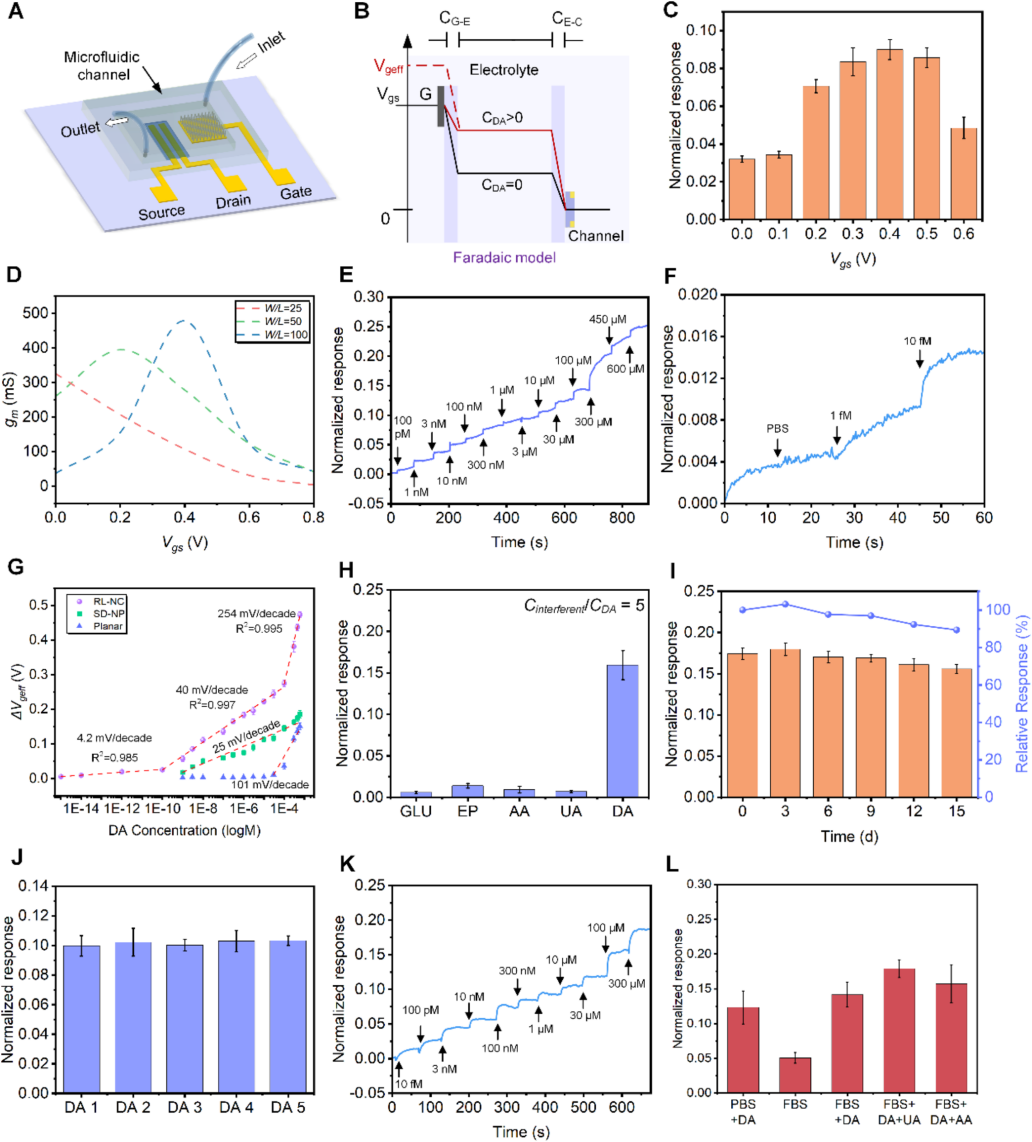
In vitro DA detection using the MSDA-OECT. (A) Schematic diagram of the MSDA-OECT platform used for in vitro DA detection. (B) Potential diagram of the OECT before and after the addition of DA. (C) NR changes with 10 μM DA at different *V*_gs_ values (0–0.6 V). (D) *g*_m_ of MSDA-OECT with different semiconductor channel width-to-length ratios (*W* = 5 mm, *L* = 50, 100, and 200 μm). (E, F) *I*_ds_ responses of the MSDA-OECT to the successive addition of different DA concentrations in PBS solution (*V*_ds_ = −0.4 V, and *V*_gs_ = 0.2 V). (G) Relationship between *V*_geff_ and DA concentration on a logarithmic scale. The dashed line indicates the linear fitting of the data. (H) Selectivity tests of the MSDA-OECT: *I*_*ds*_ responses with the addition of 100 μM DA and 500 μM interfering substances. (I) Stability tests of the MSDA-OECT: *I*_ds_ response with the addition of 100 μM DA after storage for 0, 3, 6, 9, 12, and 15 days at 4 °C, and the relative *I*_ds_ response with respect to the initial *I*_ds_. (J) Consistency tests of the MSDA-OECT: *I*_ds_ response with the addition of 10 μM DA to five MSDA-OECTs fabricated under the same conditions. (K) *I*_ds_ responses of the MSDA-OECT to successive addition of different concentrations of DA in FBS. FBS was diluted in PBS solution at a ratio of 1:10. (L) Differences in*I*_ds_responses in FBS and PBS; the addition of DA and interfering substances were 100 and 500 μM, respectively. (C, G, H, I, J, L) Error bars represent the standard error of the mean (*n* = 3).

To validate the ability of the MSDA-OECT for quantitative detection of DA, its continuous responsive behavior was recorded by adding DA to the PBS electrolyte. With the enhanced mass transfer, catalytic efficiency, and ion migration from the multiscale micropillar gate electrode, this triple-enhancement effect significantly reduces the DA detection limit of the MSDA-OECT to an exceptionally low 0.6 fM (Figure 4E,F and Supporting Section 1). Such a detection limit is 11 orders of magnitude lower than the 26.5 μM limit of the planar electrode and 6 orders of magnitude lower than the 364 pM limit of the SD-NP micropillar electrode (Supporting Section 1 and Figure S28). Compared with the DA sensor reported in the literature, the MSDA-OECT also demonstrated ultralow detection limits (Table S2). By converting *I*_ds_ into changes in *V*_geff_, the MSDA-OECT also exhibits higher sensitivity, with three linear response stages: from 1 fM to 100 pM with a slope value of 4.2 mV/decade (*R* ^2^ = 0.985); from 100 PM to 100 μM with a slope value of 40 mV/decade (*R* ^2^ = 0.997); and from 100 to 600 μM with a slope value of up to 254 mV/decade (*R* ^2^ = 0.994) (Figures 4G and S29). These results further validate the triple-enhancement effect on the enhanced sensing performance of the MSDA-OECT and demonstrate its capability for ultrasensitive DA detection.

To evaluate the MSDA-OECT’s selectivity, interfering substances, such as AA, UA, glucose (GLU), and epinephrine (EP), were added. Even with interfering substances at concentrations 5 times higher than DA, their measured value remains negligible compared to DA (Figures 4H and S30). Notably, MSDA-OECT still exhibits low responsiveness to serotonin, which is also positively charged (Figure S31). This high DA selectivity results from the combined effect of Nafion’s electrostatic repulsion of negatively charged molecules (AA and UA) and the optimized *V*_gs_, both of which facilitate DA oxidation with maximum efficiency. Since the DA detection of MSDA-OECT requires no enzyme modification, the MSDA-OECT demonstrates long-term DA detection capability and excellent stability, retaining 90% of its initial *I*_*ds*_ response after 15 days of storage at 4 °C (Figure 4I). Besides, consistency testing on five MSDA-OECTs fabricated using the same steps shows a low NR change variance of 1.56% (Figure 4J), indicating high repeatability of the temperature-induced self-assembly AJP method. To better mimic the real physiological environment, the MSDA-OECT tested in fetal bovine serum (FBS) maintained a low detection limit of 10 fM (Figure 4K), demonstrating excellent potential for clinical detection. Moreover, the MSDA-OECT’s DA detection ability in FBS differs by less than 5% compared to PBS, which exhibits its anti-interference ability with proteins in serum (Figures 4L and S32). This demonstrates the feasibility of the quantitative calibration of DA in PBS, significantly simplifying the calibration process for DA detection in a real physiological environment.

### Wireless, Real-Time, and Highly Sensitive Monitoring of DA In Vivo

Real-time, highly sensitive in vivo monitoring of DA has long been a challenge.^48–50^ Here, the ultralow detection limit, high stability, and selectivity exhibited by MSDA-OECT demonstrate its potential for real-time DA monitoring in vivo. To achieve in situ signal processing and wireless data transmission, a wearable MSDA-OECT sensing system was further developed by integrating the MSDA-OECT with an FPCB and a battery power supply (Figure 5A,B). The biocompatible poly­(3,4-ethlenedioxythiophene): poly­(styrenesulfonate) (PEDOT:PSS) and rGO ensure the MSDA-OECT’s biosafety,^51–53^ with over 95% cell survival rate after 24 h of coculture with L929 rat fibroblasts (Figure 5C). To fit within the confined space of the brain while ensuring effective DA sensing, a 150 μm-high RL-NC micropillar array was used as a gate electrode, and PDMS blocks were positioned on both sides to protect these micropillars from damage. The MSDA-OECT was surgically implanted into the rabbit brain and secured with sutures, while the FPCB was attached to the back using a biotape (Figure 5D,E). The transfer characteristic curve and the derived *g*_m_ of the MSDA-OECT captured in the cerebrospinal fluid (CF) show high consistency with that in PBS (Figure S33), further demonstrating the feasibility of establishing an in vitro calibration curve in PBS.

**Figure 5:**
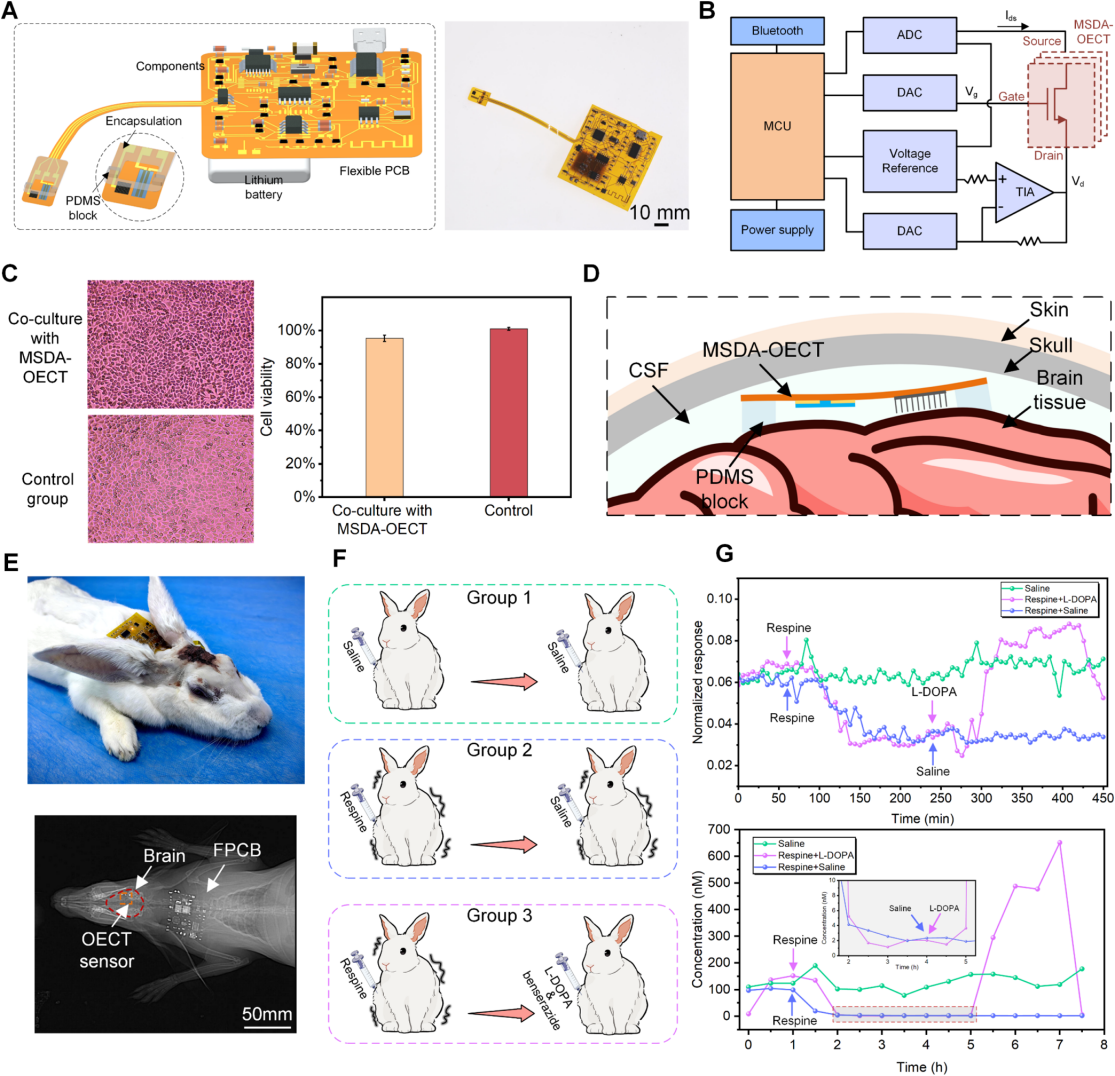
Wireless, real-time, and highly sensitive monitoring of DA in rabbits. (A) Schematic illustration and optical image of the MSDA-OECT wearable sensing system. (B) System-level block and schematic diagram of the electronic circuit. (C) Left: Microscopic images of L929 rat fibroblasts cocultured with the control group and MSDA-OECT extract for 24 h. Cells cocultured with the MSDA-OECT extract show no signs of cell lysis and a decline in proliferation. Right: Comparison of cell activity between the control group and cells cocultured with the extract. Error bars represent the standard error of the mean (*n* = 3). (D) Schematic diagram of MSDA-OECT inside the rabbit brain. (E) Optical and X-ray images of the MSDA-OECT wearable sensing system implanted in a rabbit brain. (F) Schematic diagram of the biological experimental scheme of the Parkinson’s disease model: group 1, the normal group; group 2, the only reserpine-induced group; and group 3, the reserpine-induced group following treatment with L-DOPA and benserazide. (G) Three groups continuously monitored the *I*_ds_ changes and DA concentration fluctuation in the rabbit brain for 7.5 h.

Parkinson’s disease (PD), characterized by impaired motor function, muscle rigidity, and tremor, is closely linked to decreased DA secretion.^54,55^ Thus, the PD animal model was used to investigate the monitoring feasibility of MSDA-OECT in vivo. Reserpine was utilized to construct the PD animal model due to its inhibitory effect on vesicular monoamine transporters, resulting in reduced DA secretion.^56^ Afterward, the combination of levodopa (L-DOPA) and benserazide was administered to the control group to promote DA secretion and temporarily relieve PD symptoms. Figure 5F illustrates the entire process of the animal model study, in which six rabbits were divided into three groups, each implanted with MSDA-OECT for continuous monitoring over 7.5 h. The *I*_ds_ of the MSDA-OECT was collected every 5 min for 60 s (Figure S34), and the continuous response of the NR is shown in Figure 5G. To more intuitively reflect the changes in DA in the brain, the NR changes were converted into the corresponding DA concentration according to the in vitro PBS quantitative calibration curve of DA (Figures 5G and S35). Compared with the control group (group 1) injected with normal saline only, the rabbits injected with reserpine (groups 2, 3) exhibit typical PD symptoms such as tremor and bradykinesia, and the DA concentration in the CF drops from 100 nM in the normal state to 2 nM. After the combined administration of L-DOPA and benserazide (group 3), the biological activity of rabbits gradually recovered, and the DA concentration recovered to 500 nM. These results demonstrate that the MSDA-OECT wearable sensing system enables real-time, wireless, and highly sensitive monitoring of DA fluctuations in biological fluids, providing crucial diagnostic and therapeutic information for a deeper understanding and treatment of DA-related diseases.

## Conclusions

In summary, we developed an MSDA-OECT with a nanocluster-assembled multiscale micropillar gate electrode for ultrasensitive DA detection. An aerosol jet 3D printing method with temperature-induced self-assembly was developed to precisely fabricate multiscale micropillar structures, and the formation mechanism of these multiscale structures was investigated. The MSDA-OECT facilitates a high sensitivity of 254 mV/decade and an ultralow DA detection limit of 0.6 fM through the triple-enhancement effect of multiscale micropillar: (1) the 3D geometry and nanoclusters of multiscale micropillar enhance mass transfer, facilitating the capture and accumulation of DA, with improvements of up to 14.4 times compared to the planar gate structure; (2) nanoclusters increase rGO loading and local electric field, boosting the catalytic reaction of DA; and (3) the increased surface area of the multiscale micropillar improves the ion migration and gate modulation, enabling the high amplification of the OECT. By integration with an FPCB, a wearable sensing system was developed to enable real-time, wireless, and highly sensitive DA monitoring in the brain of a PD rabbit model, validating the feasibility of highly sensitive DA monitoring by MSDA-OECT in real physiological environments. Furthermore, by modifying the multiscale gate electrodes, the multiscale OECT can be readily utilized for ultrasensitive detection of various biomarkers, demonstrating significant potential for applications in clinical diagnostics and precision medicine.

## Methods

### Fabrication of the Multiscale Micropillar Electrodes

A schematic diagram of the micropillar electrode fabrication process is shown in Figure 2A. All of the micropillar structures were fabricated using a self-assembly aerosol jet 3D printer integrated with a two-stage heating device and Ag NP ink (BroadCON-INK550; BroadTeko Co., Ltd.). N_2_ served as the carrier gas and sheath gas to transport and focus the aerosol microdroplets, respectively. During the micropillar printing process, the carrier gas flow rate was set between 20 and 30 sccm, the sheath gas flow rate was maintained at 40 sccm, and a print head with a 150 μm inner diameter was used at a printing speed of 1 mm/s. The micropillars were printed in a circular trajectory, with their height determined by the number of printing passes. The self-assembly process of aerosol microdroplets was controlled by adjusting the preheater temperature between 30 and 50 °C, while the deposition state of the micropillar was regulated by setting the substrate temperature between 100 and 160 °C. The Ag NP ink concentration was diluted from 30 to 5 wt % with deionized water to study its effect on the NC diameter. The printed multiscale micropillars were sintered at 300 °C for 30 min after printing to remove the binder and further sinter the NP and NC.

### Fully Printed Fabrication of MSDA-OECT and the Three-Electrode Test System

The source, drain, and planar gate electrodes of the MSDA-OECT were printed onto a PI film (thickness: 25 μm) by using Ag NP ink. A 400 μm print head was selected, with carrier and sheath gas flow rates of 40 sccm and 80 sccm, respectively, and a printing speed of 10 mm/s. The substrate temperature was maintained at 80 °C during printing. After printing, the electrodes were sintered at 300 °C for 30 min to ensure excellent conductivity. Then, the above multiscale micropillar fabrication process was applied to print SD-NP/NC micropillars and RL-NC micropillar arrays onto the planar gate electrode. The rGO solution (1 mg/mL; Xianfeng Nanomaterial Technology Co., Ltd.) was added to 5% Nafion (5.0 wt % in water and 1-propanol; Aladdin) and sonicated for 1 h. The rGO and Nafion mixture solution was then printed onto the surface of the gate electrode by aerosol jet 3D printing to ensure uniform coverage. During the printing process, a 400 μm print head was used with carrier and sheath flow rates of 60 and 80 sccm, respectively. The printing speed was 10 mm/s, and the substrate temperature was maintained at 80 °C. Then, the material was dried at 100 °C for 1 h. Aqueous PEDOT:PSS (PH1000, Clevios) supplemented with 5% glycerin (Macklin) and 1% 3-glycidyloxypropyltrimethoxysilane (Macklin) was magnetically stirred for 1 h and then printed as the semiconductor channel. A 1 mm print head was selected, with carrier and sheath gas flow rates of 60 and 80 sccm, respectively, and a printing speed of 10 mm/s. The printed semiconductor channel was sintered at 140 °C for 30 min.

In the three-electrode test system, the working, reference, and counter electrodes were printed with Ag NP ink using the same printing parameters as those for the electrodes in the MSDA-OECT. Additionally, the printing of micropillars on the working electrode, as well as rGO and Nafion, followed the same printing steps as in the MSDA-OECT.

### Microscopic Characterization and Reliability Testing of the Multiscale Micropillar Electrode

Scanning electron microscopy (SEM) was used to characterize the morphology of the multiscale micropillar electrodes. Elemental composition and mappings of the micropillar after printing rGO and Nafion were obtained by using EDX elemental analysis. For reliability testing, we first characterized the micropillar structure using SEM. The micropillars were then exposed to a 2 m/s water flow from a microfluidic pump syringe for 5 min. After drying, SEM was employed to observe and compare the structural differences in the same areas.

### Electrochemical Characterization of the Multiscale Micropillar Electrode in a Three-Electrode System

Electrochemical characterization was conducted by using a CHI660 electrochemical workstation (Shanghai Chenhua Instrument Co., Ltd.). A 200 μL PBS solution (50 mM, pH 7.4, Macklin) was added as the electrolyte. CV measurements were conducted at a scan rate of 0.1 V/s over a range of −0.8 to 0 V, with a pulse amplitude of 50 mV.

EASA was calculated from the capacitance measured by CV at various scan rates within the non-Faradaic range, using 0.1 M KHCO_3_ (Macklin) as the electrolyte, with Pt as the counter electrode and Ag/AgCl as the reference electrode. The EASA was calculated using the following equation

3 $$\frac{I_{a}-I_{c}}{2}=\mathrm{Cv}$$

where *C* is the capacitance, *I*_a_ is the anodic current, *I*_c_ is the cathode current, and *v* is the scan rate. An aerosol jet printing planar electrode was used as the standard reference.

### MSDA-OECT Characterization and In Vitro Detection of DA

The MSDA-OECT was characterized using Keithley source meters (Keithley 2602), which used *V*_ds_ and *V*_gs_ while measuring *I*_ds_. The PDMS microchannel, formed by replica molding, was placed above the semiconductor channel layer and gate electrode. A 200 μL PBS buffer solution (50 mM, pH 7.4) was added to the PDMS microchannel to obtain the MSDA-OECT’s output and transfer characteristic curves. The DA quantification in vitro was measured at *V*_gs_ = 0.2 V and *V*_ds_ = −0.4 V. The DA PBS solution or DA FBS solution was injected into the microchannel via a microfluidic pump syringe at a flow rate of 30 mL/min. After complete replacement of the solution within the microchannel, the injection was halted to ensure a static testing environment.

FBS (Aladdin) was diluted in PBS solution at a ratio of 1:10. Based on the normalized *I*_ds_ response and the transfer characteristic curve, the recorded *I*_ds_ can be converted into *V*_geff_. The conversion procedure involves the following steps. (1) The normalized response corresponding to the DA at different concentrations is obtained by using eq 2. (2) The transfer characteristic curve is then used to establish the correlation between the DA concentration and *V*_geff_, from which the change in Δ*V*_geff_ is derived.

### In Vitro Cytotoxicity Evaluation

The MSDA-OECT was sterilized by drying at 120 °C for 30 min. It was then mixed with Minimum Essential Medium (MEM) containing 10% FBS, shaken at 37 °C for 24 h, and centrifuged at 800 rpm for 5 min to obtain the extract. Rat fibroblast L929 cells were inoculated into a 96-well plate and cultured for 24 h to form a semiconfluent monolayer. The cells were then cocultured with the control group (MEM culture medium containing 10% FBS) and the MSDA-OECT extract for 24 h. The cell growth and cell viability in each group were observed under a microscope.

### MSDA-OECT Wearable Sensing System

An MSDA-OECT wearable sensing system with control and wireless data transmission capabilities was developed. It was integrated by an FPCB and an MSDA-OECT and encapsulated with a PDMS (Sylgard 184, Dow Corning) film by selective coating. PDMS blocks (2 × 3 mm^2^) were positioned on both sides of the sensing unit to protect the RL-NC micropillar array during implantation. The microcontroller unit (MCU, ATMega328P-AU, MICROCHIP) was programmed to output *V*_ds_ and *V*_gs_ using a digital-to-analog converter and to collect *I*_ds_ through an analog-to-digital converter. The entire wireless system was powered by two micro-lithium batteries (3.7 V, 50 mA). The voltage regulator module and reverse chip supplied power to the operational amplifier, MCU, and other components. The collected signals were transmitted to a PC or mobile phone via a Bluetooth module (KT6368A, Jieli).

### In Vivo Dopamine Sensing in a Rabbit Model

Animal procedures were performed in agreement with the institutional guidelines of Beijing Tonghe Litai Biotechnology Co., Ltd. The experimental protocols were reviewed and approved by the Institutional Animal Care and Use Committee at Beijing Tonghe Litai Biotechnology Co., Ltd. (Protocol No. SND2024071). Six New Zealand rabbits (weighing 2–3 kg) were acclimated for 5 days before surgery. Before implantation, the rabbits were anesthetized with the compound ketamine. The hair on the rabbits’ heads was shaved, and a craniotomy (3 mm wide by 10 mm long) was performed. The MSDA-OECT of the wearable sensing system was implanted into the brain and secured with dental cement. The wound was then disinfected and sutured to complete the procedure. The FPCB of the wearable sensing system was fixed to the dorsal region of the rabbit using biotape, and the rabbit was immobilized using a restraining box to prevent damage to the wearable sensing system. The rabbits were allowed to recover for 12 h before the DA monitoring experiment was commenced. The six rabbits were divided into three groups: (1) normal rabbit group, (2) reserpine-induced Parkinson’s animal model, (3) reserpine-induced Parkinson’s animal model with combined administration of L-DOPA and benserazide. The rabbits were continuously monitored for 7.5 h, and *I*_ds_ values were recorded every 5 min for 60 s. In the first injection step, the normal rabbit group (group 1) was injected with saline (containing 1% glacial acetic acid). Four rabbits (groups 2 and 3) were intraperitoneally injected with reserpine (1 mg/kg, dissolved in 1% glacial acetic acid). After monitoring for 3 h, groups 1 and 2 were injected with saline (containing 1% glacial acetic acid) intraperitoneally, and group 3 was injected with a combination of levodopa (200 mg/kg) and benserazide (50 mg/kg), and *I*_*ds*_ values were monitored again for 3.5 h.

### Simulations

The COMSOL Multiphysics software (version 5.5, COMSOL Inc., Burlington, MA, USA) was used to perform finite element simulations. In the electrochemical module of COMOSOL, the electrochemical reaction was set as Fe­(CN)_6_^3–^ + e^–^ ↔ Fe­(CN)_6_^4–^. At the electrode boundary, Fe­(CN)_6_^4–^ was oxidized to Fe­(CN)_6_^3–^, wherein the Fe­(CN)_6_^4–^ concentration was uniform and the Fe­(CN)_6_^3–^ concentration was zero. The electroanalytical Butler–Volmer equation was used to evaluate the redox current.

## Supplementary Material







## Data Availability

All relevant data that support the findings of this study are presented in the manuscript and Supporting Information file. Source data are available from the corresponding author upon reasonable request.

## Notes

Infrared thermography image of the aerosol jet 3D printing system; transformation process of nanoclusters; SEM images of nanoclusters at different concentrations of ink; SEM images of SD-NC micropillar arrays; relationship between the number of printing passes and height of micropillar structures; EASA characterization of different electrode structures; COMOSOL simulation of diffusion streamlines for the SD-NP micropillar and SD-NC micropillar electrode; images of the fabrication process of DA OECT; real-time *I*_ds_ responses with the addition of interfering substances; real-time monitoring of *I*_ds_ in the rabbit brain; comparison of diffusion coefficient with varied electrodes; comparison of the detection performance of DA sensors from the previously reported works and this work (PDF)
Fabrication of multiscale micropillars via temperature-induced self-assembly aerosol jet printing (Movie S1) (MP4)
Fully printed fabrication of MSDA-OECT via aerosol jet printing (Movie S2) (MP4)

## References

[1] Ou Z. J., Pan J., Tang S. H., Duan D. P., Yu D. F., Nong H. Q., Wang Z. (2021). Global Trends
in the Incidence, Prevalence, and Years Lived with
Disability of Parkinson’s Disease in 204 Countries/Territories
from 1990 to 2019. Front. Public Health.

[2] Zhang Z. F., Jackson S. L., Gillespie C., Merritt R., Yang Q. H. (2023). Depressive
Symptoms and Mortality among Us Adults. JAMA
Netw. Open.

[3] Peritogiannis V., Ninou A., Samakouri M. (2022). Mortality in Schizophrenia-Spectrum
Disorders: Recent Advances in Understanding and Management. Healthcare.

[4] Woody C. A., Ferrari A. J., Siskind D. J., Whiteford H. A., Harris M. G. (2017). A Systematic Review and Meta-Regression of the Prevalence
and Incidence of Perinatal Depression. J. Affect.
Disord..

[5] Ikemoto S. (2007). Dopamine Reward
Circuitry: Two Projection Systems from the Ventral Midbrain to the
Nucleus Accumbens-Olfactory Tubercle Complex. Brain Res. Rev..

[6] Robinson D. L., Hermans A., Seipel A. T., Wightman R. M. (2008). Monitoring Rapid
Chemical Communication in the Brain. Chem. Rev..

[7] Klein M. O., Battagello D. S., Cardoso A. R., Hauser D. N., Bittencourt J. C., Correa R. G. (2019). Dopamine: Functions, Signaling, and Association with
Neurological Diseases. Cell. Mol. Neurobiol..

[8] Matt S. M., Gaskill P. J. (2020). Where Is
Dopamine and How Do Immune Cells See It?:
Dopamine-Mediated Immune Cell Function in Health and Disease. J. Neuroimmune Pharm..

[9] Du J., Yue R. R., Ren F. F., Yao Z. Q., Jiang F. X., Yang P., Du Y. K. (2014). Novel Graphene
Flowers Modified Carbon
Fibers for Simultaneous Determination of Ascorbic Acid, Dopamine and
Uric Acid. Biosens. Bioelectron..

[10] Yao Y., Huang W., Chen J. H., Liu X. X., Bai L. B., Chen W., Cheng Y. H., Ping J. F., Marks T. J., Facchetti A. (2023). Flexible and
Stretchable Organic Electrochemical Transistors
for Physiological Sensing Devices. Adv. Mater..

[11] Wang L., Yue X. P., Sun Q. Z., Zhang L. R., Ren G. Z., Lu G., Yu H. D., Huang W. (2022). Flexible Organic Electrochemical
Transistors for Chemical and Biological Sensing. Nano Res..

[12] Song J. J., Liu H., Zhao Z. Y., Lin P., Yan F. (2024). Flexible Organic Transistors
for Biosensing: Devices and Applications. Adv.
Mater..

[13] Parlak O., Keene S. T., Marais A., Curto V. F., Salleo A. (2018). Molecularly
Selective Nanoporous Membrane-Based Wearable Organic Electrochemical
Device for Noninvasive Cortisol Sensing. Sci.
Adv..

[14] Chen J. H., Huang W., Zheng D., Xie Z. Q., Zhuang X. M., Zhao D., Chen Y., Su N., Chen H. M., Pankow R. M. (2022). Highly Stretchable Organic Electrochemical
Transistors with Strain-Resistant Performance. Nat. Mater..

[15] Liao C. Z., Zhang M., Niu L. Y., Zheng Z. J., Yan F. (2014). Organic Electrochemical
Transistors with Graphene-Modified Gate Electrodes for Highly Sensitive
and Selective Dopamine Sensors. J. Mater. Chem.
B.

[16] Mak C. H., Liao C. Z., Fu Y., Zhang M., Tang C. Y., Tsang Y. H., Chan H. L. W., Yan F. (2015). Highly-Sensitive Epinephrine
Sensors Based on Organic Electrochemical Transistors with Carbon Nanomaterial
Modified Gate Electrodes. J. Mater. Chem. C.

[17] Zhang L. J., Wang G. H., Wu D., Xiong C., Zheng L., Ding Y. S., Lu H. B., Zhang G. B., Qiu L. Z. (2018). Highly
Selective and Sensitive Sensor Based on an Organic Electrochemical
Transistor for the Detection of Ascorbic Acid. Biosens. Bioelectron..

[18] Wang Y., Xiong C., Qu H., Chen W., Ma A. J., Zheng L. (2017). Highly Sensitive Real-Time Detection
of Tyrosine Based on Organic
Electrochemical Transistors with Poly-(Diallyldimethylammonium Chloride),
Gold Nanoparticles and Multi-Walled Carbon Nanotubes. J. Electroanal. Chem..

[19] Wu X. Y., Feng J. Y., Deng J., Cui Z. C., Wang L. Y., Xie S. L., Chen C. R., Tang C. Q., Han Z. Q., Yu H. B. (2020). Fiber-Shaped
Organic Electrochemical Transistors for
Biochemical Detections with High Sensitivity and Stability. Sci. China Chem..

[20] Soleymani L., Fang Z. C., Lam B., Bin X. M., Vasilyeva E., Ross A. J., Sargent E. H., Kelley S. O. (2011). Hierarchical Nanotextured
Microelectrodes Overcome the Molecular Transport Barrier to Achieve
Rapid, Direct Bacterial Detection. ACS Nano.

[21] Ali M. A., Hu C. S., Yuan B., Jahan S., Saleh M. S., Guo Z. T., Gellman A. J., Panat R. (2021). Breaking the Barrier
to Biomolecule Limit-of-Detection Via 3d Printed Multi-Length-Scale
Graphene-Coated Electrodes. Nat. Commun..

[22] Valera A. E., Nesbitt N. T., Archibald M. M., Naughton M. J., Chiles T. C. (2019). On-Chip
Electrochemical Detection of Cholera Using a Polypyrrole-Functionalized
Dendritic Gold Sensor. ACS Sens..

[23] Saleh M. S., Hu C. S., Panat R. (2017). Three-Dimensional
Microarchitected
Materials and Devices Using Nanoparticle Assembly by Pointwise Spatial
Printing. Sci. Adv..

[24] Saleh M. S., Li J., Park J., Panat R. (2018). 3d Printed
Hierarchically-Porous
Microlattice Electrode Materials for Exceptionally High Specific Capacity
and Areal Capacity Lithium Ion Batteries. Addit.
Manuf..

[25] Herbert R., Lim H. R., Rigo B., Yeo W. H. (2022). Fully Implantable
Wireless Batteryless Vascular Electronics with Printed Soft Sensors
for Multiplex Sensing of Hemodynamics. Sci.
Adv..

[26] Saleh M. S., Ritchie S. M., Nicholas M. A., Gordon H. L., Hu C. S., Jahan S., Yuan B., Bezbaruah R., Reddy J. W., Ahmed Z. (2022). Cmu Array: A 3d Nanoprinted,
Fully Customizable High-Density Microelectrode Array Platform. Sci. Adv..

[27] Zhou X. Z., Zhang L. W., Wang Y., Zhao S., Zhou Y., Guo Y. R., Wang Y. M., Liang J., Chen H. W. (2023). Aerosol
Jet Printing of Multi-Dimensional Oect Force Sensor with High Sensitivity
and Large Measuring Range. Adv. Mater. Technol..

[28] Chen X. L., Lawrence J. M., Wey L. T., Schertel L., Jing Q. S., Vignolini S., Howe C. J., Kar-Narayan S., Zhang J. Z. (2022). 3d-Printed Hierarchical Pillar Array Electrodes for
High-Performance Semi-Artificial Photosynthesis. Nat. Mater..

[29] Smith B. N., Ballentine P., Doherty J. L., Wence R., Hobbie H. A., Williams N. X., Franklin A. D. (2024). Aerosol Jet Printing
Conductive 3d
Microstructures from Graphene without Post-Processing. Small.

[30] Ali M. A., Hu C. S., Jahan S., Yuan B., Saleh M. S., Ju E. G., Gao S. J., Panat R. (2021). Sensing of Covid-19
Antibodies in Seconds Via Aerosol Jet Nanoprinted Reduced-Graphene-Oxide-Coated
3d Electrodes. Adv. Mater..

[31] Karim A., Yasser M., Ahmad A., Natsir H., Wahab A. W., Fauziah S., Taba P., Pratama I., Rosalin, Rajab A. (2024). A Review:
Progress and Trend Advantage of Dopamine Electrochemical Sensor. J. Electroanal. Chem..

[32] Tığ G. A. (2017). Development
of Electrochemical Sensor for Detection of Ascorbic Acid, Dopamine,
Uric Acid and L-Tryptophan Based on Ag Nanoparticles and Poly­(L-Arginine)-Graphene
Oxide Composite. J. Electroanal. Chem..

[33] Jia L. P., Zhou Y. X., Jiang Y. M., Zhang A. H., Li X., Wang C. M. (2016). A Novel Dopamine
Sensor Based on Mo Doped Reduced Graphene
Oxide/Polyimide Composite Membrane. J. Alloy.
Compd..

[34] Ji W., Wu D. Q., Tang W., Xi X., Su Y. Z., Guo X. J., Liu R. L. (2020). Carbonized Silk Fabric-Based Flexible
Organic Electrochemical Transistors for Highly Sensitive and Selective
Dopamine Detection. Sens. Actuators, B.

[35] Basiri S., Mehdinia A., Jabbari A. (2018). Green Synthesis
of Reduced Graphene
Oxide-Ag Nanoparticles as a Dual-Responsive Colorimetric Platform
for Detection of Dopamine and Cu^2+^. Sens. Actuators, B.

[36] Li Z. X., Chen F. B., Zhu N., Zhang L. M., Xie Z. (2023). Tip-Enhanced
Sub-Femtomolar Steroid Immunosensing Via Micropyramidal Flexible Conducting
Polymer Electrodes for at-Home Monitoring of Salivary Sex Hormones. ACS Nano.

[37] Ali M. A., Tabassum S., Wang Q. G., Wang Y. F., Kumar R., Dong L. (2018). Integrated Dual-Modality
Microfluidic Sensor for Biomarker Detection
Using Lithographic Plasmonic Crystal. Lab Chip.

[38] Liu M., Pang Y. J., Zhang B., De Luna P., Voznyy O., Xu J. X., Zheng X. L., Dinh C. T., Fan F. J., Cao C. H. (2016). Enhanced
Electrocatalytic CO_2_ Reduction
Via Field-Induced Reagent Concentration. Nature.

[39] Liu P., Chen B., Liang C. W., Yao W. T., Cui Y. Z., Hu S. Y., Zou P. C., Zhang H., Fan H. J., Yang C. (2021). Tip-Enhanced Electric
Field: A New Mechanism Promoting Mass Transfer
in Oxygen Evolution Reactions. Adv. Mater..

[40] Sharma R., Ali M. A., Selvi N. R., Singh V. N., Sinha R. K., Agrawal V. V. (2014). Electrochemically Assembled Gold
Nanostructures Platform:
Electrochemistry, Kinetic Analysis, and Biomedical Application. J. Phys. Chem. C.

[41] Zhou Y. G., Wan Y., Sage A. T., Poudineh M., Kelley S. O. (2014). Effect of Microelectrode
Structure on Electrocatalysis at Nucleic Acid-Modified Sensors. Langmuir.

[42] Tarabella G., Santato C., Yang S. Y., Iannotta S., Malliaras G. G., Cicoira F. (2010). Effect of the Gate
Electrode on the Response of Organic
Electrochemical Transistors. Appl. Phys. Lett..

[43] Bernards D. A., Malliaras G. G. (2007). Steady-State
and Transient Behavior of Organic Electrochemical
Transistors. Adv. Funct. Mater..

[44] Rivnay J., Inal S., Salleo A., Owens R. M., Berggren M., Malliaras G. G. (2018). Organic Electrochemical Transistors. Nat. Rev. Mater..

[45] Deng Y. P., Qi H., Ma Y., Liu S. B., Zhao M. Y., Guo Z. H., Jie Y. S., Zheng R., Jing J. Z., Chen K. T. (2022). A Flexible
and Highly Sensitive Organic Electrochemical Transistor-Based
Biosensor for Continuous and Wireless Nitric Oxide Detection. Proc. Natl. Acad. Sci. U.S.A..

[46] Xie K., Wang N. X., Lin X. D., Wang Z. X., Zhao X., Fang P. L., Yue H. B., Kim J. W., Luo J., Cui S. Y. (2020). Organic Electrochemical Transistor Arrays for Real-Time
Mapping of Evoked Neurotransmitter Release in Vivo. eLife.

[47] Tang H., Yan F., Lin P., Xu J. B., Chan H. L. W. (2011). Highly Sensitive
Glucose Biosensors Based on Organic Electrochemical Transistors Using
Platinum Gate Electrodes Modified with Enzyme and Nanomaterials. Adv. Funct. Mater..

[48] Li J. X., Liu Y. X., Yuan L., Zhang B. B., Bishop E. S., Wang K. C., Tang J., Zheng Y. Q., Xu W. H., Niu S. M. (2022). A Tissue-Like
Neurotransmitter Sensor for the Brain
and Gut. Nature.

[49] Wu G. F., Zhang N. N., Matarasso A., Heck I., Li H. J., Lu W., Phaup J. G., Schneider M. J., Wu Y. X., Weng Z. Y. (2022). Implantable
Aptamer-Graphene Microtransistors for Real-Timemonitoring
of Neurochemical Release in Vivo. Nano Lett..

[50] Zhou L., Yang R. J., Li X. R., Dong N., Zhu B. Y., Wang J. J., Lin X. Y., Su B. (2023). Cof-Coated Microelectrode
for Space-Confined Electrochemical Sensing of Dopamine in Parkinson’s
Disease Model Mouse Brain. J. Am. Chem. Soc..

[51] Zhang S. M., Chen Y. H., Liu H., Wang Z. T., Ling H. N., Wang C. S., Ni J. H., Saltik B. C., Wang X. C., Meng X. (2020). Room-Temperature-Formed Pedot:Pss Hydrogels Enable
Injectable, Soft, and Healable Organic Bioelectronics. Adv. Mater..

[52] Wang J., Li Q., Li K. C., Sun X., Wang Y. Z., Zhuang T. T., Yan J. J., Wang H. (2022). Ultra-High Electrical Conductivity
in Filler-Free Polymeric Hydrogels toward Thermoelectrics and Electromagnetic
Interference Shielding. Adv. Mater..

[53] Kim Y. K., Kim M. H., Min D. H. (2011). Biocompatible Reduced
Graphene Oxide
Prepared by Using Dextran as a Multifunctional Reducing Agent. Chem. Commun..

[54] Senel M., Dervisevic E., Alhassen S., Dervisevic M., Alachkar A., Cadarso V. J., Voelcker N. H. (2020). Microfluidic Electrochemical
Sensor for Cerebrospinal Fluid and Blood Dopamine Detection in a Mouse
Model of Parkinson’s Disease. Anal. Chem..

[55] Powell D., Threlkeld A. J., Fang X., Muthumani A., Xia R. P. (2012). Amplitude- and Velocity-Dependency of Rigidity Measured
at the Wrist in Parkinson’s Disease. Clin. Neurophysiol..

[56] Leão A. H., Sarmento-Silva A. J., Santos J. R., Ribeiro A. M., Silva R. H. (2015). Molecular,
Neurochemical, and Behavioral Hallmarks of Reserpine as a Model for
Parkinson’s Disease: New Perspectives to a Long-Standing Model. Brain Pathol..

