# Supplementary material for: Self-Assembly of 3D-Printed Multiscale Micropillar-Based Organic Electrochemical Transistors for Ultrasensitive Dopamine Sensing
Source: ACS Nano. 2025 Aug 5;19(46):39615–27. doi: 10.1021/acsnano.5c02784 (PMC12659440; doi:10.1021/acsnano.5c02784)
Supplement: Supplementary file 1 [file nn5c02784_si_001.pdf]

Supporting Information for

A Self-assembly 3D Printed Multiscale Micropillar-based Organic Electrochemical Transistor for Ultra-sensitive Dopamine Sensing

Xinzhao Zhou[1], Liwen Zhang[1], Shengbin Zhang[1], Jing Liang[1], Ke Zhang[1], Zehui Zhao[2], Song Zhao[1], Yan Wang[1], Yurun Guo[1], Deyuan Zhang[1], Lei Jiang[3], Huawei Chen[1,4*]*

[1]School of Mechanical Engineering and Automation, Beihang University, Beijing, 100191, China

[2]College of Mechanical and Transportation Engineering, China University of Petroleum, Beijing, 102249, China

[3]Laboratory of Bioinspired Smart Interface Science, Technical Institute of Physics and Chemistry, Chinese Academy of Sciences, Beijing, 100190, China

[4]Beijing Advanced Innovation Centre for Biomedical Engineering, Beihang University, Beijing, 100191, China

[*]Corresponding author. Email: chenhw75@buaa.edu.cn; lwzhang@buaa.edu.cn

Supplementary Section 1: Calculation of Detection Limit for MSDA-OECT

The detection limit of MSDA-OECT was evaluated using a method reported in literature.[1-2]

Limit of blank (LOB) = Mean of signal (blank sample) + 1.645 * (Standard deviation of blank sample) (1)

Limit of detection of the signal (Y_{LOD}) = LOB + 1.645 * (Standard deviation of DA at low concentration) (2)

C_{LOD} = (Y_{LOD} - C)/slope of the OECT calibration (3)

Here, "C" is the intercept of the calibration curve of the OECT. Both $I_{ds}^{C_{DA}}$ and I_{ds}^{0} were obtained at the stable state response of the OECT. Specifically, the $I_{ds}^{C_{DA}}$ and I_{ds}^{0} are defined as the average of three data points recorded at 2-second intervals in reverse chronological order, starting from the moment when the next concentration of DA was added.

For OECT with planar electrode, the mean of the blank signal and the standard deviation are 2.83 mV and 0.32 mV [n = 3], respectively (Figure S29). From Eq. (1), the LOB is calculated as 3.36 mV. Mean signal at the lowest concentration (30 μM of DA) is 11.67 mV, with a standard deviation of 1.42 mV [n = 3]. From Eq. (2), Y_{LOD} is calculated as 5.70 mV. The OECT with planar electrode calibration equation is Y_{LOD} (mV) = 101.14 * Log [C_{DA}] + 468.58 (Figure 4G). This gives C = 468.58 and slope of the OECT calibration curve as 101.14. Subsite the above results into Eq. (3), the C_{LOD} of the OECT with planar electrode is 26.5 μM.

For OECT with SD-NP micropillar, the mean of the blank signal and the standard deviation are 2.55 mV and 1.12 mV [n = 3], respectively (Figure S29). From Eq. (1), the LOB is calculated as 4.39 mV. Mean signal at the lowest concentration (1 nM of DA) is 18.23 mV, with a standard deviation of 1.66 mV [n = 3]. From Eq. (2), Y_{LOD}

is calculated as 7.12 mV. The OECT with SD-NP micropillar calibration equation is Y_{LOD} (mV) = 25 * Log [C_{DA}] + 243.1 (Figure 4G). This gives C = 243.1 and slope of the OECT calibration curve as 25. Subsite the above results into Eq. (3), the C_{LOD} of the OECT with SD-NP micropillar is 364 pM.

For OECT with RL-NC micropillar (MSDA-OECT), the mean of the blank signal and the standard deviation are 1.5 mV and 0.92 mV [n = 3], respectively (Figure S29). From Eq. (1), the LOB is calculated as 3.01 mV. Mean signal at the lowest concentration (1 fM of DA) is 6.21 mV, with a standard deviation of 1.26 mV [n = 3]. From Eq. (2), Y_{LOD} is calculated as 5.08 mV. The MSDA-OECT calibration equation is Y_{LOD} (mV) = 4.2 * Log [C_{DA}] + 68.94 (Figure 4G). This gives C = 68.94 and slope of the OECT calibration curve as 4.2. Subsite the above results into Eq. (3), the C_{LOD} of the MSDA-OECT is 0.6 fM.

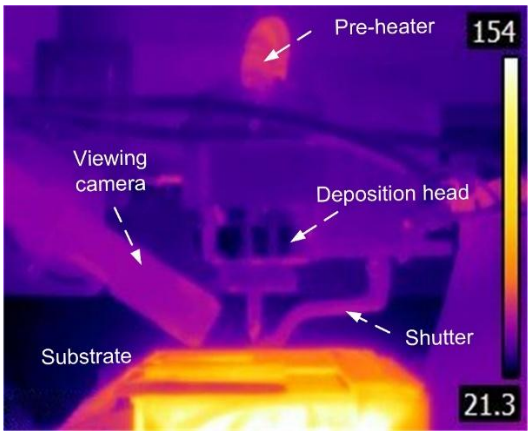

Figure S1. Infrared thermography image of the aerosol jet 3D printing system with temperature-induced self-assembly. The pre-heater and heated substrate construct the two-stage heating process.

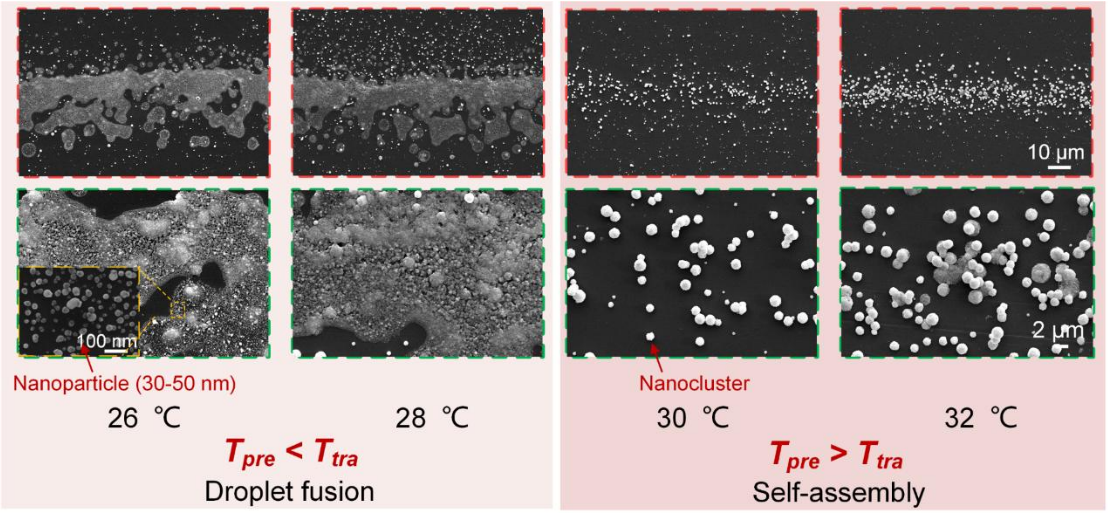

Figure S2. SEM images of printed tracks at different preheating temperatures and a carrier gas flow rate of 20 sccm. The printing speed is set to 12 mm/s to easily observe the states of the nanoparticles and nanoclusters. When the $T_{pre} < T_{tra}$, a significant amount of solvent remains in the microdroplets, causing them to merge with each other on the substrate. The inset exhibits numerous dispersed Ag nanoparticles. As preheating temperature increases, the solvent of the microdroplet gradually evaporates, and once the $T_{pre} > T_{tra}$, the nanoparticles self-assemble into nanoclusters.

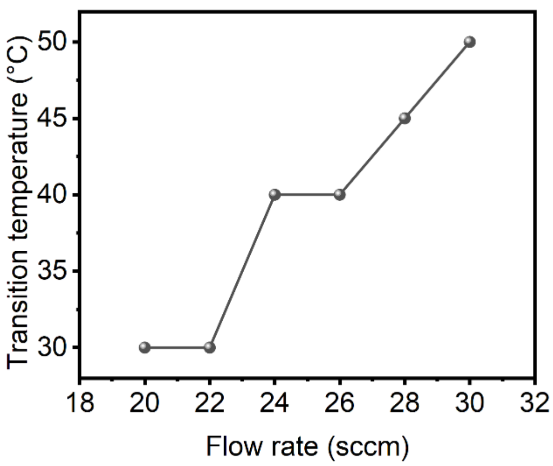

Figure S3. The relationship between T_{tra} and carrier gas flow rate.

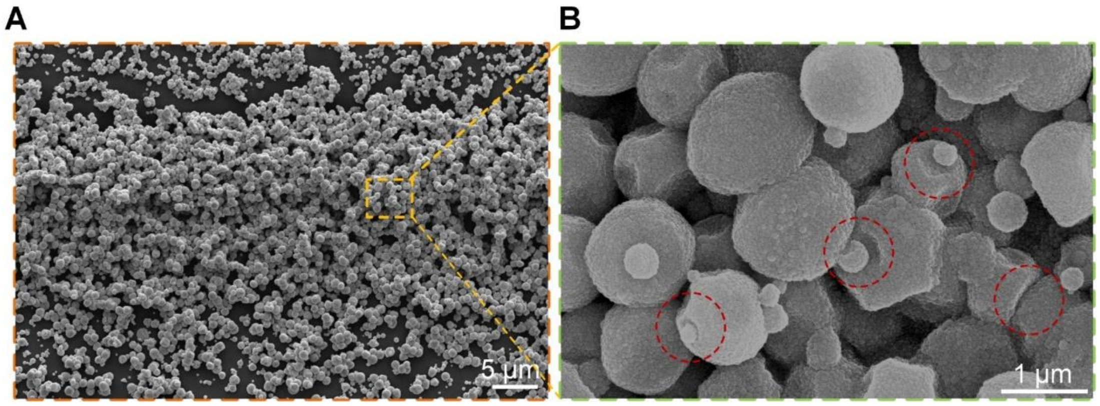

Figure S4. SEM images of printed track. (A) SEM image of printed track at the printing speed of 1mm/s and preheating temperature of 40 ℃. (B) SEM image obtained at higher magnification shows the nanoclusters do not merge with each other even after undergoing collisions.

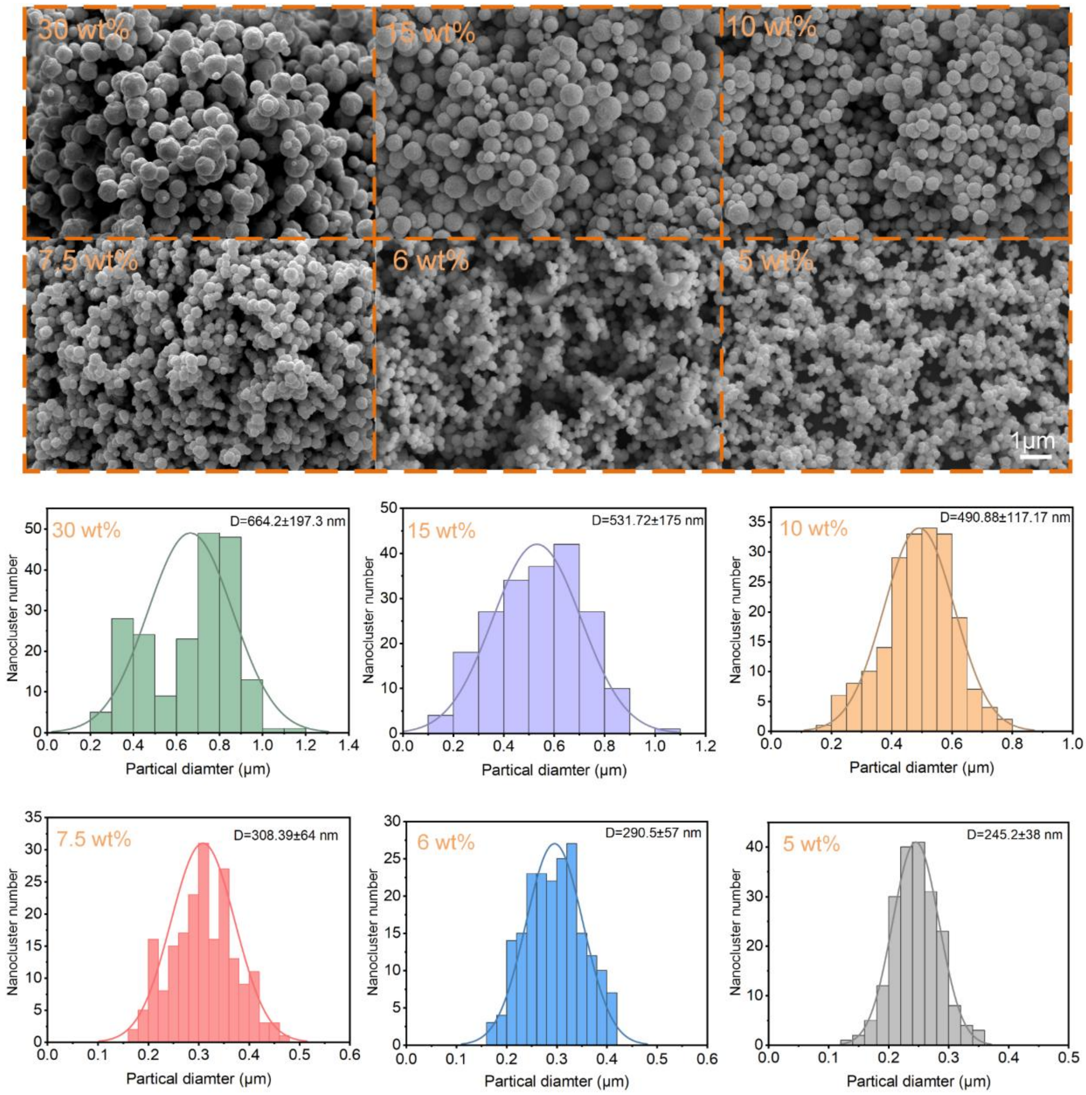

Figure S5. Size statistical distribution and SEM of nanoclusters under different ink concentrations. At each concentration, 200 nanoclusters are counted to get the size distribution.

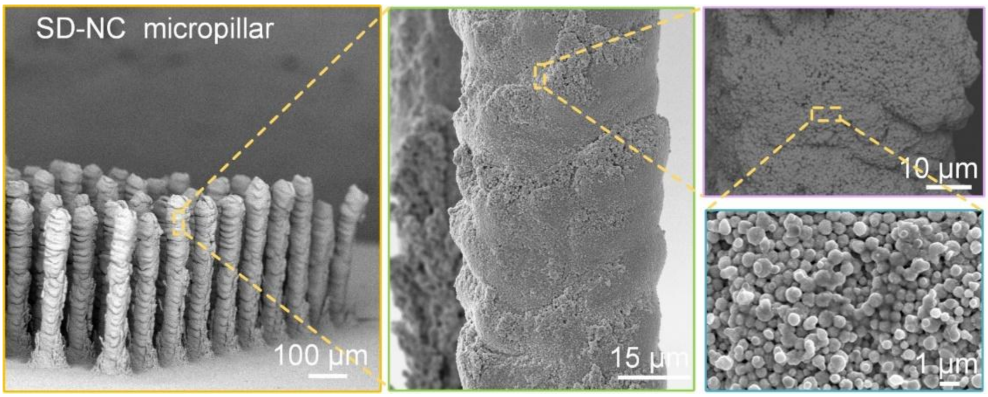

Figure S6. SEM images of SD-NC micropillars array (450 μm height, 60 μm diameter and 200 μm center distance).

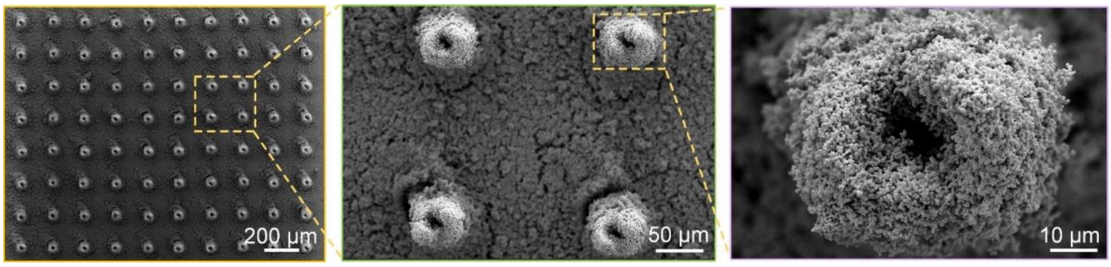

Figure S7. Top-down SEM images of RL-NC micropillars array.

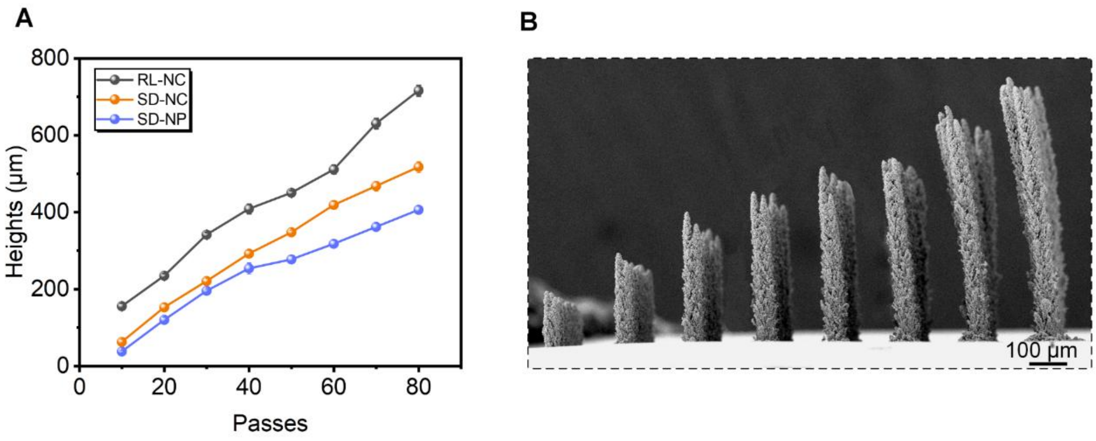

Figure S8. Relationship between the number of printing passes and height of different types of micropillar structures. (A) Relationship between the number of printing passes and height of different types of micropillar structures. Error bars represent the standard error of the mean (n = 3). (B) SEM image of the RL-NC micropillars with different heights.

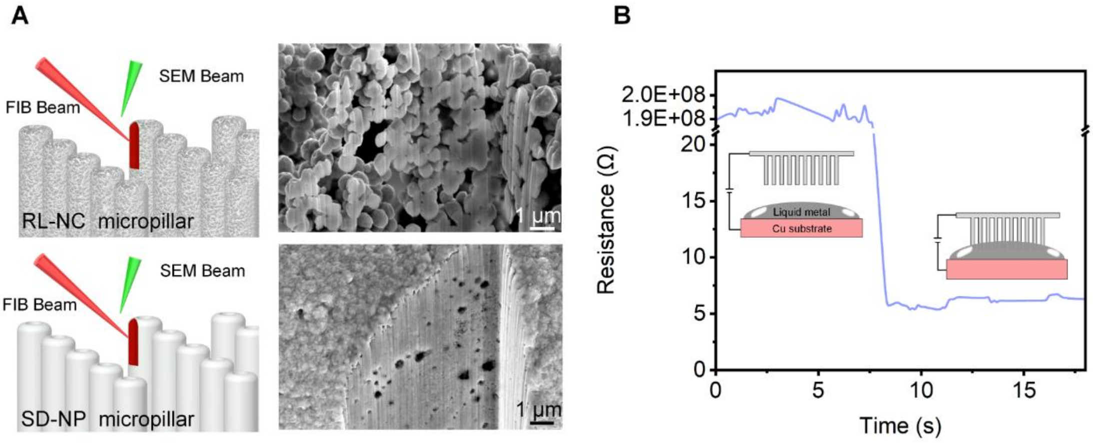

Figure S9. Cross-section characterization and conductivity test of RL-NC micropillars array. (A)Schematic diagrams and SEM images of SD-NP micropillar and RL-NC micropillar etched by focused ion beam (FIB). (B) Conductivity test of RL-NC micropillars array. Liquid metal coated on the copper substrate as the other electrode for conductivity test.

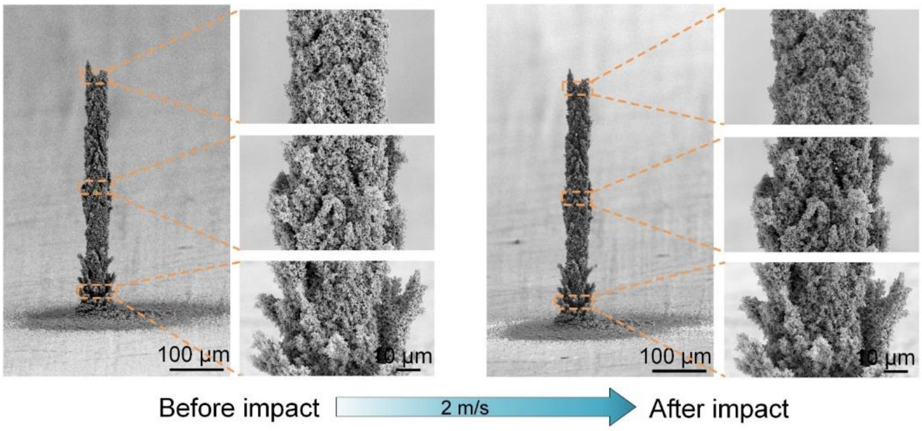

Figure S10. SEM images of the RL-NC micropillar before and after being impacted by the 2 m/s water flow. Insets show the detailed comparison of different parts.

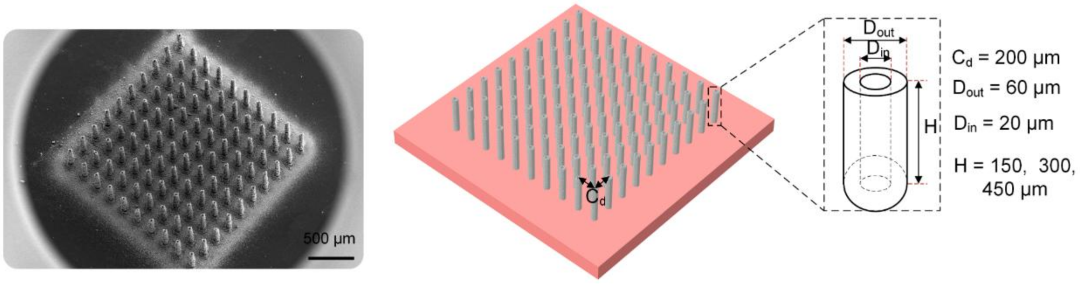

Figure S11. SEM image and schematic diagram of micropillars array electrode.

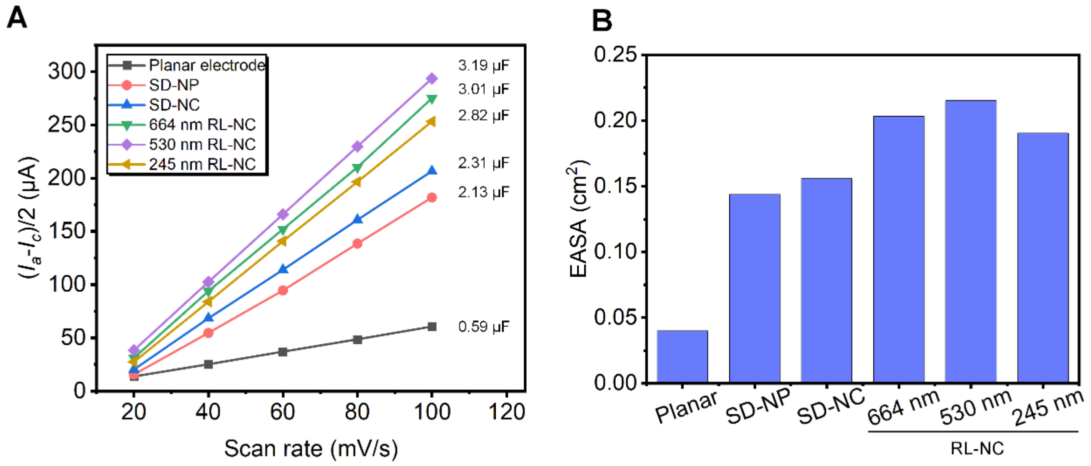

Figure S12. EASA characterization of different electrode structures. (A) Summary of the capacitance measurements used to calculate the EASA of planar electrode (fabricated by aerosol jet printing), SD-NP/NC micropillars (height: 450 μm) and RL-NC assembled from different NC diameters (height: 450 μm). (B) Summary of measured EASAs of above electrodes.

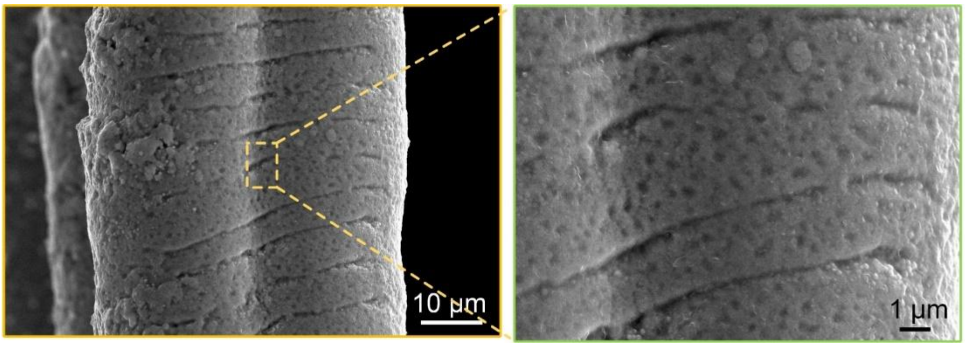

Figure S13. SEM images of rGO wrapped on the surface of SD-NP micropillar.

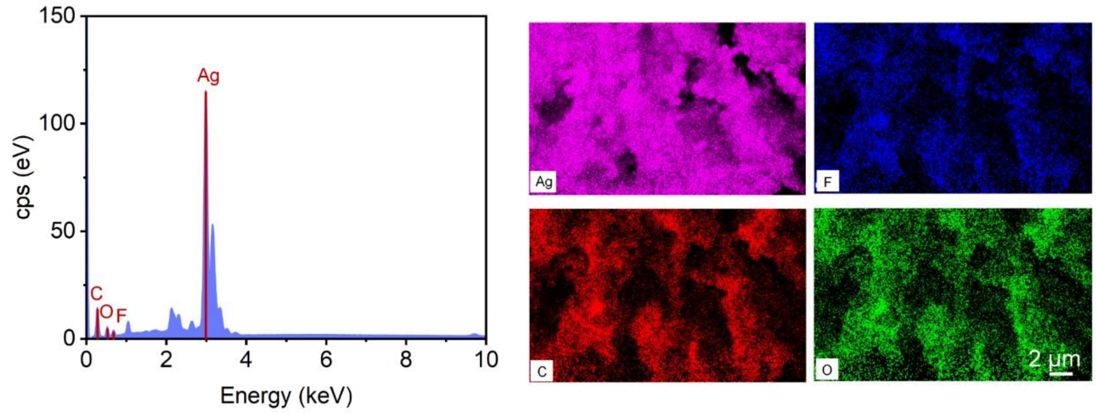

Figure S14. EDX elements energy spectrum and elemental distribution after the rGO and Nafion loaded on the surface of RL-NC micropillar.

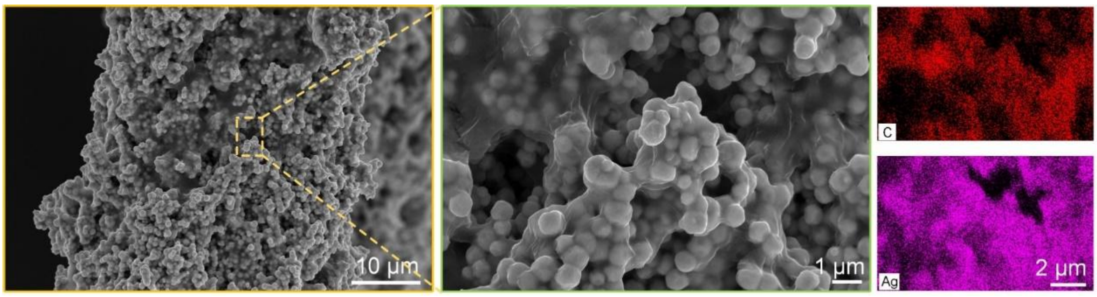

Figure S15. SEM images and the elemental distribution of the RL-NC micropillar loaded with only rGO. By comparing, the deposition of Nafion does not alter the surface morphology of RL-NC micropillar.

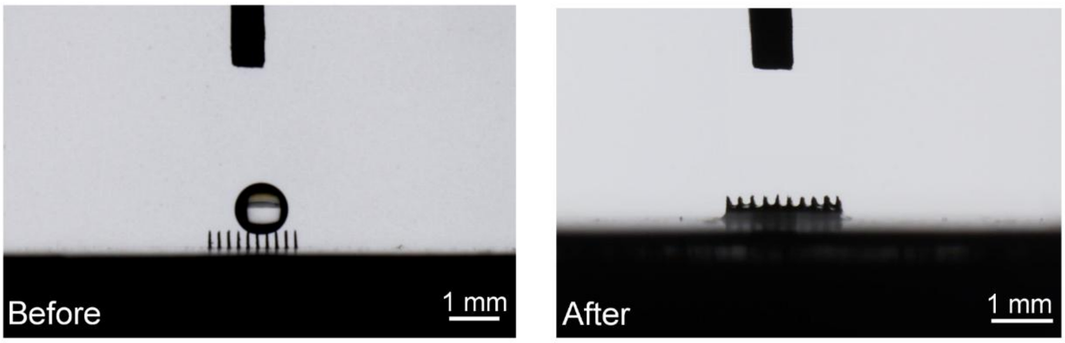

Figure S16. Changes in wettability of RL-NC micropillars array before and after loading with rGO. Before loading with rGO, the RL-NC micropillars array exhibits superhydrophobic properties, limiting the contact between the DA solution and the RL-NC micropillars array electrode, which is not conducive to the detection of DA.

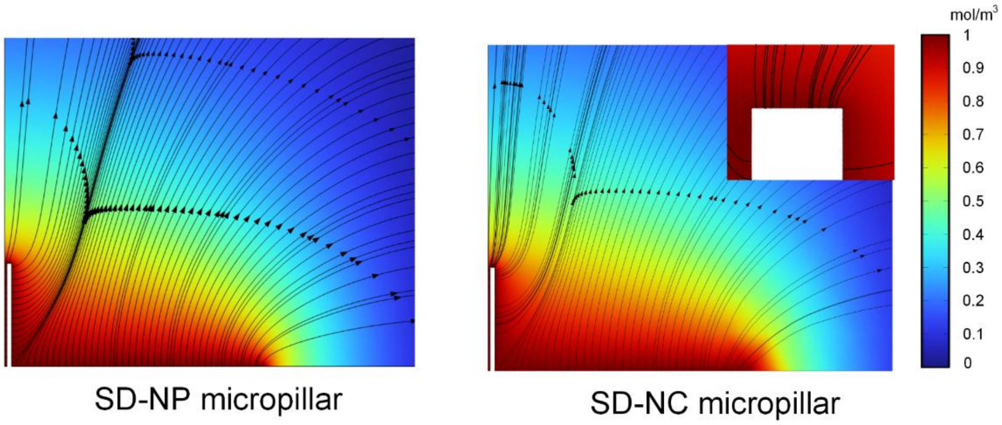

Figure S17. COMOSOL simulation of diffusion streamlines for half of the single SD-NP micropillar and SD-NC micropillar electrode. Inset is the magnified view of the diffusion streamlines around the top electrode.

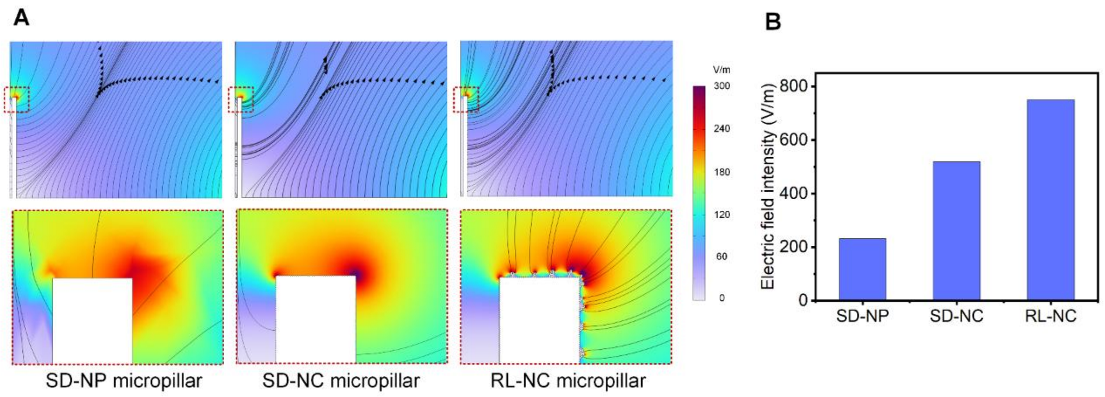

Figure S18. COMOSOL simulation for Electric field distribution of different types of micropillar structures (half of a single micropillar). (A) Electric field distribution on different electrode surface. (B) Electric field intensity at the tip of the nanocluster on the SD-NC micropillar and RL-NC micropillars, compared to the surface of the SD-NP micropillar electrode, at a voltage of 0.2 V. The finite element simulation of the RL-NC micropillar results exhibits that the local electric field is significantly enhanced due to the tip discharge effect of the nanoclusters.

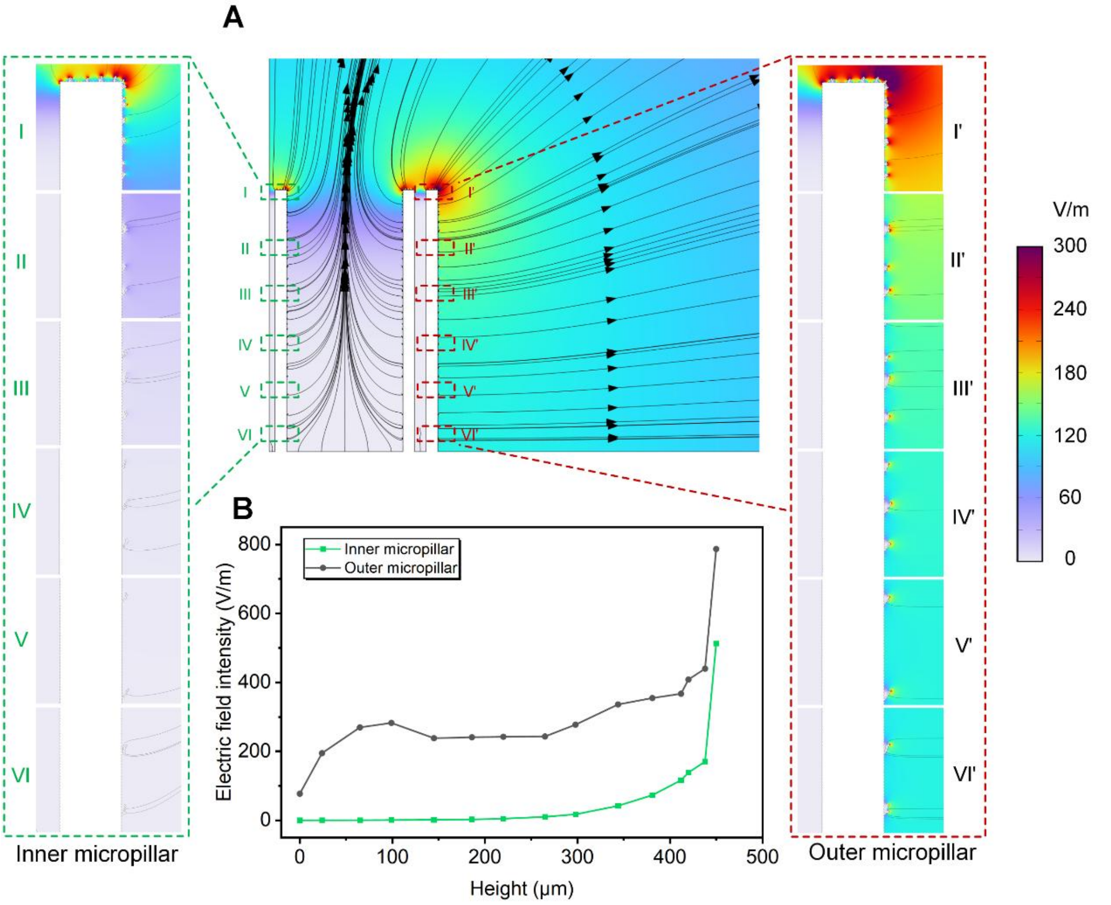

Figure S19. Simulation of electric field distribution of both inner and outer RL-NC micropillars (A whole pillar and half of a single pillar). (A) Electric field distribution of both inner and outer micropillars. Magnified views on the left and right illustrate the distribution of the electric field at the nanocluster tips at varying heights on the inner and outer micropillars. (B) Electric field intensity at the nanocluster tips at different heights on the inner and outer micropillars.

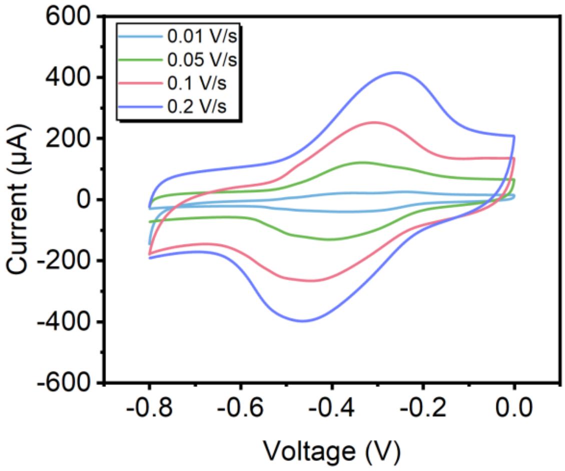

Figure S20. CV response of the RL-NC micropillar array electrode under the scan rate from 10 to 200 mV/s.

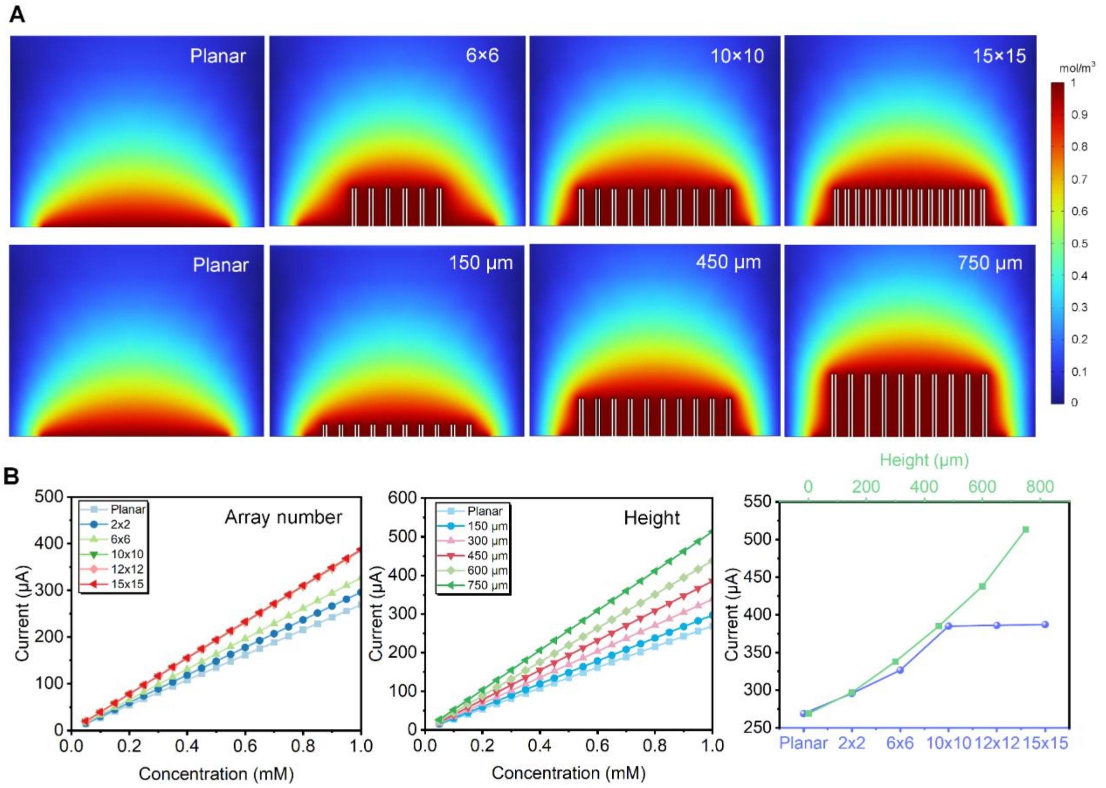

Figure S21. COMSOL simulations of RL-NC micropillar with different heights and array numbers. (A) Concentration profiles of diffusing analyte at the central plane for different arrays and heights of the RL-NC micropillars array electrodes. (B) COMSOL simulations of RL-NC micropillars array electrodes with varying array number configurations and heights at different analyte concentrations.

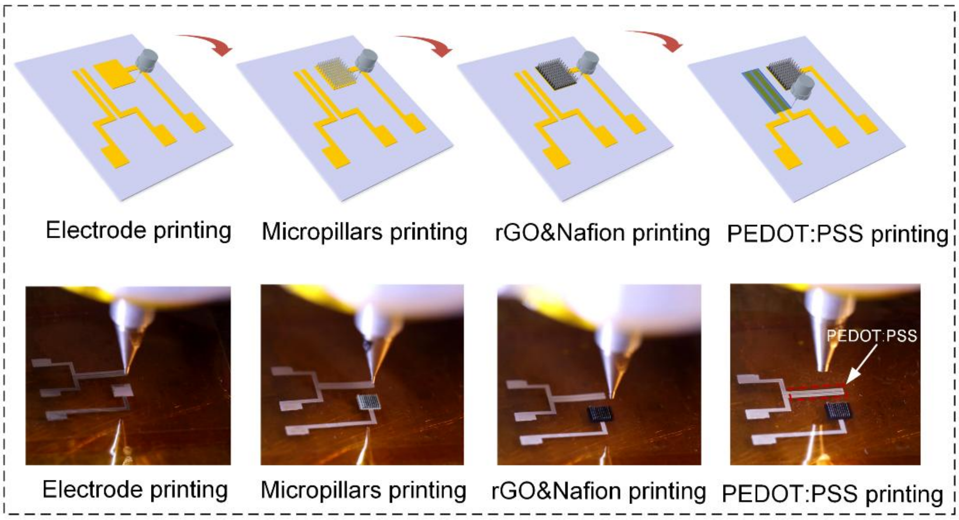

Figure S22. Schematic and images of the fabrication process of DA OECT using fully aerosol jet 3D printing method. As the typical p-type semiconductor material, the conducting polymer PEDOT:PSS was chosen as the semiconductor channel material in this study.

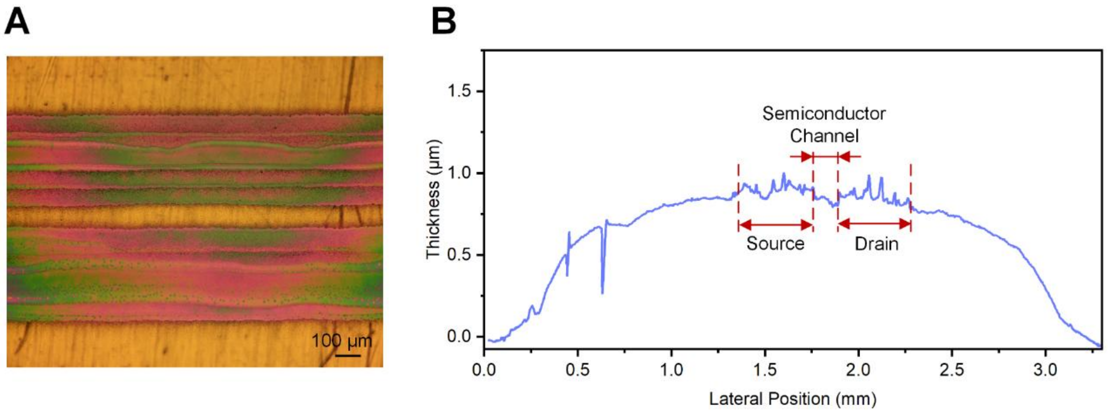

Figure S23. Characterization of the semiconductor channel layer. (A) Image of the semiconductor channel layer printed using AJP (W = 5 mm, L = 100 μm). (B) Characterization of the semiconductor channel layer thickness, which exhibits the excellent uniformity.

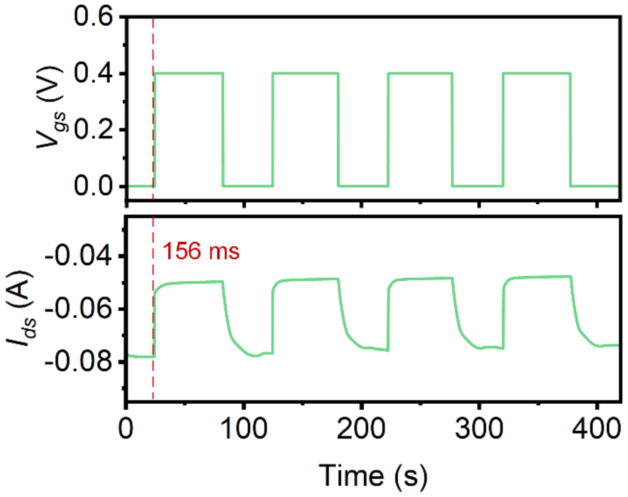

Figure S24. Response characteristic of the MSDA-OECT. The MSDA-OECT exhibits a fast response time of 156 ms.

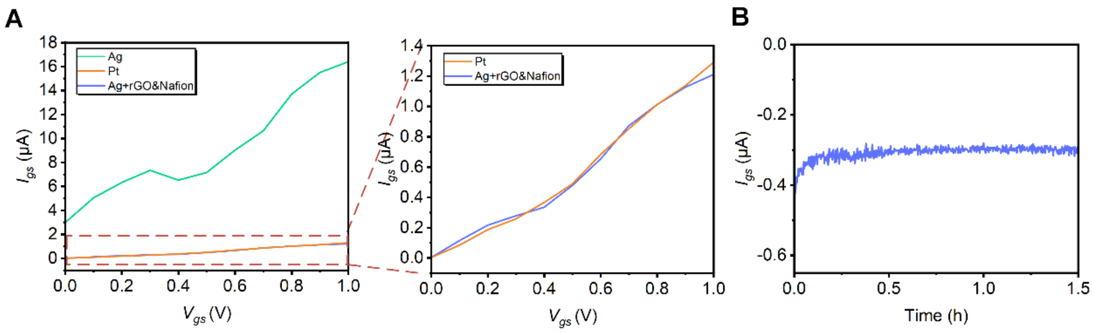

Figure S25. Characterization of the I_{gs} of the MSDA-OECT. (A) I_{gs} of the MSDA-OECT with and without the rGO and Nafion loaded on the multiscale micropillar gate electrode and I_{gs} of the OECT with Pt gate electrode (V_{ds} = -0.4V). It is known that Pt electrode does not react with Cl^-. The multiscale micropillar gate electrode with rGO and Nafion loaded exhibits a similar I_{gs} to Pt gate electrode, indicating that rGO and Nafion effectively isolate the reaction between Ag and Cl^-. (B) Long-term monitoring of the I_{gs} of the MSDA-OECT with rGO and Nafion loaded (V_{ds} = -0.4V, V_{gs} = 0.2V).

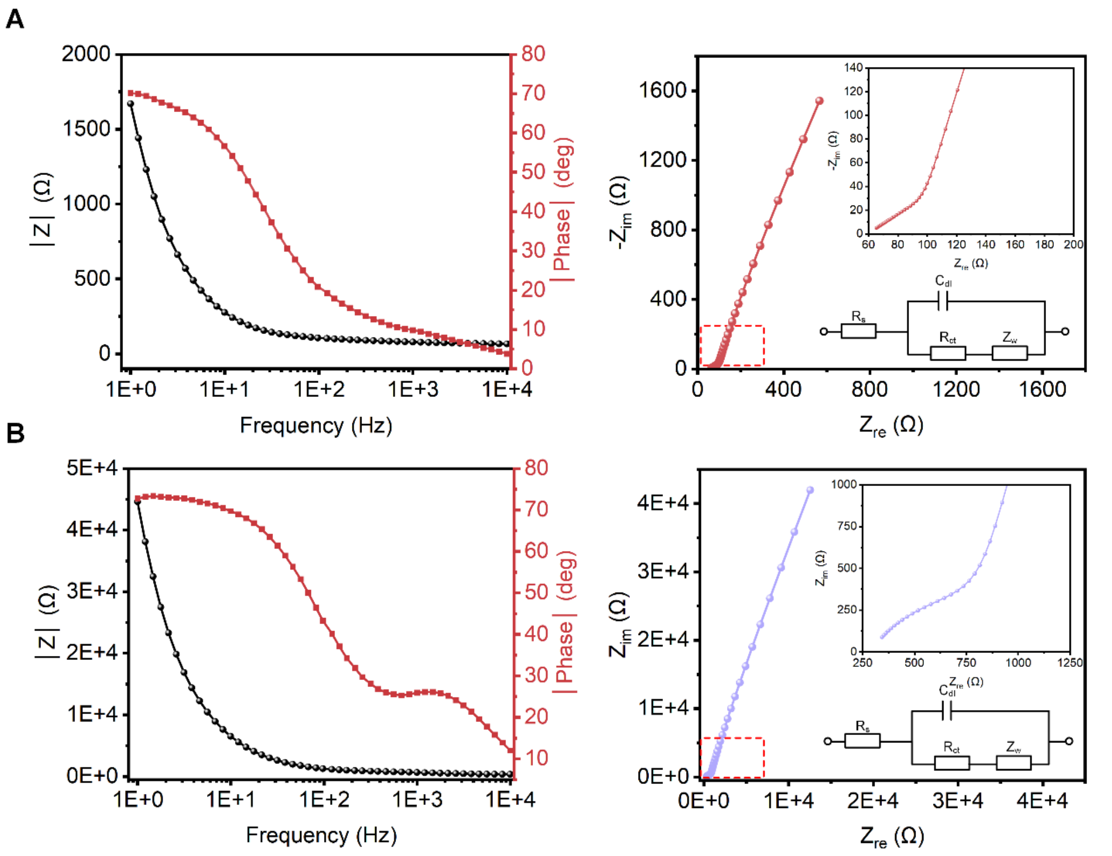

Figure S26. EIS of RL-NC micropillar gate electrode and semiconductor channel. (A) Bode and Nyquist plots of the RL-NC micropillar with 530 nm NC. Fitting parameters: R_s = 61 Ω, R_{ct} = 16.5 Ω, C_{dl} = 77 μF. (B) Bode and Nyquist plots of semiconductor channel. Fitting parameters: R_s = 292 Ω, R_{ct} = 890 Ω, C_{dl} = 3.1 μF. The EIS experiments were performed using a 5 mV amplitude over a frequency range of 1-10000Hz in PBS (50 mM, PH 7.4) containing 1 mM equimolar $[Fe (CN_6)]^{3-/4-}$. Ag/AgCl electrode was used as the reference electrode, and Pt electrode served as the counter electrode.

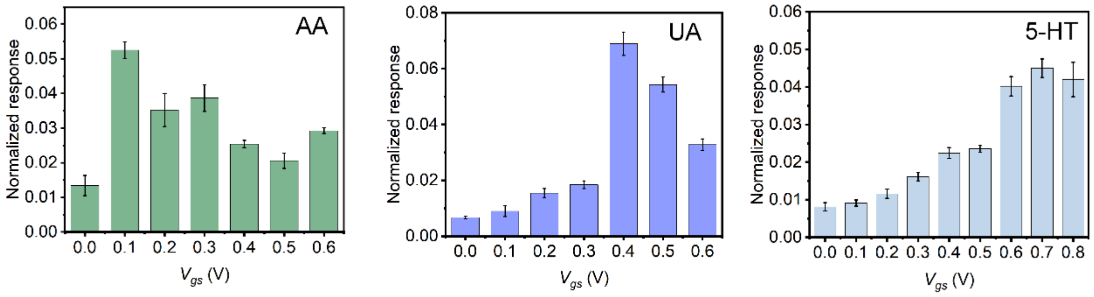

Figure S27. NR changes of the I_{ds} with the addition of AA, UA and 5-HT under different V_{gs}. The test is performed using the gate electrode without the Nafion covering. Error bars represent the standard error of the mean (n = 3).

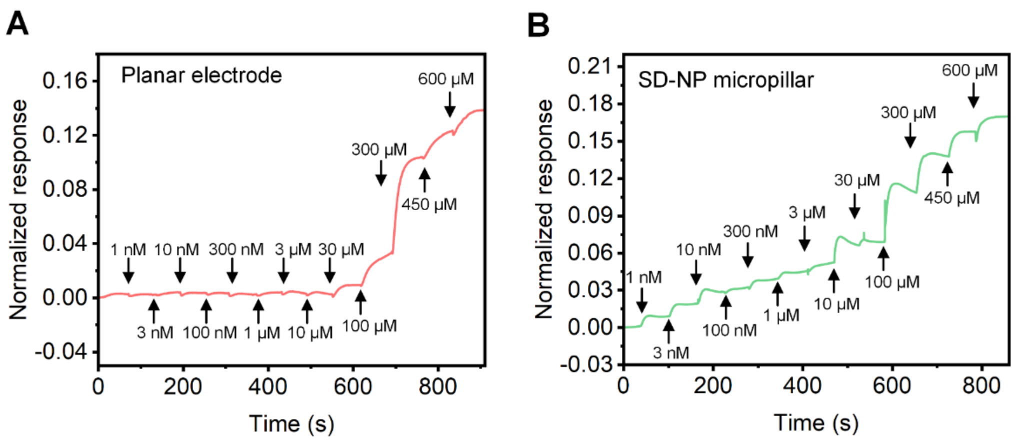

Figure S28. The I_{ds} responses of (A) DA OECT with planar gate electrode and (B) DA OECT with SD-NP micropillar array to the successive addition of different concentration DA in PBS solution (V_{ds} = -0.4 V, V_{gs} = 0.2 V).

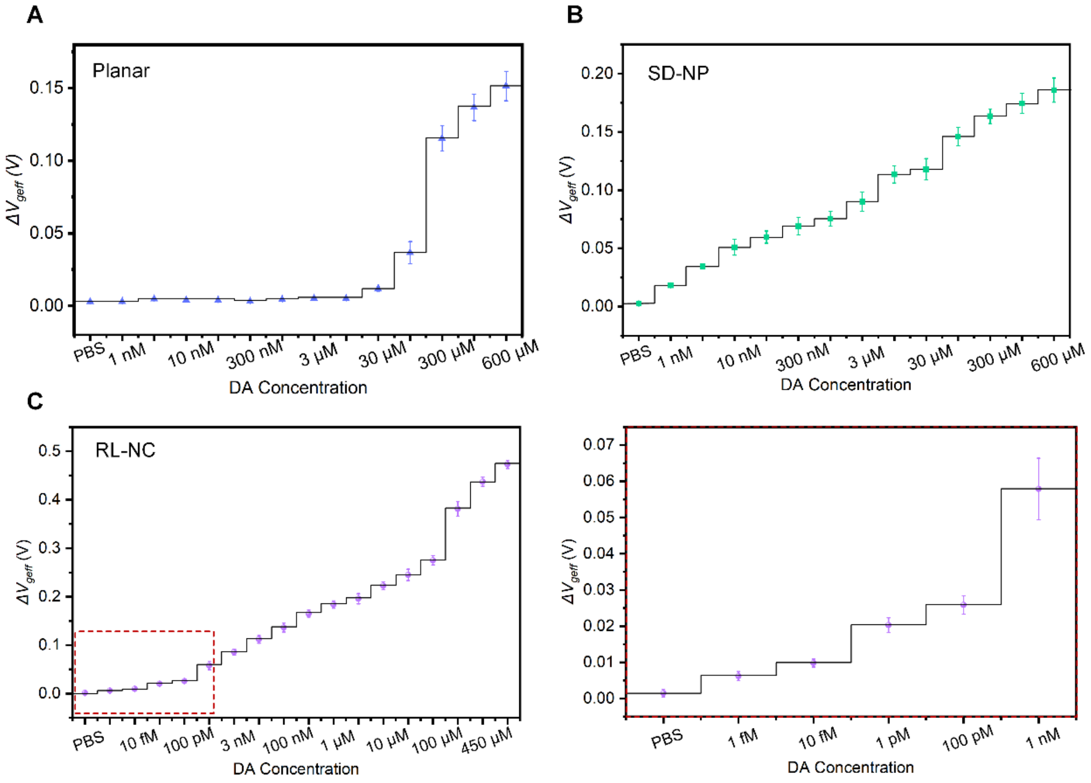

Figure S29. Relationship between V_{geff} and varying DA concentrations for OECTs with planar, SD-NP micropillar and RL-NC micropillar electrode. Error bars represent the standard error of the mean ($n = 3$).

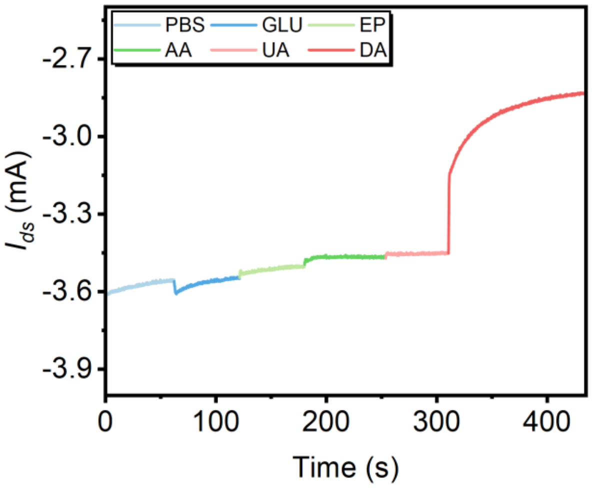

Figure S30. Real-time I_{ds} responses of MSDA-OECT with the addition of successive amounts of interfering substances. The concentrations of the DA and interfering substances are 100 μM and 500 μM, separately.

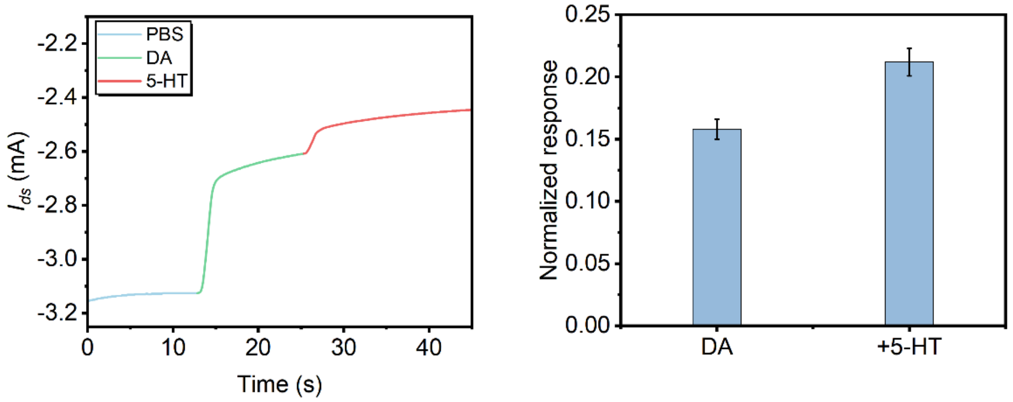

Figure S31. Real-time I_{ds} responses and NR changes of MSDA-OECT with the addition of serotonin at V_{gs} = 0.2 V. The concentrations of the DA and serotonin are 100 μM.

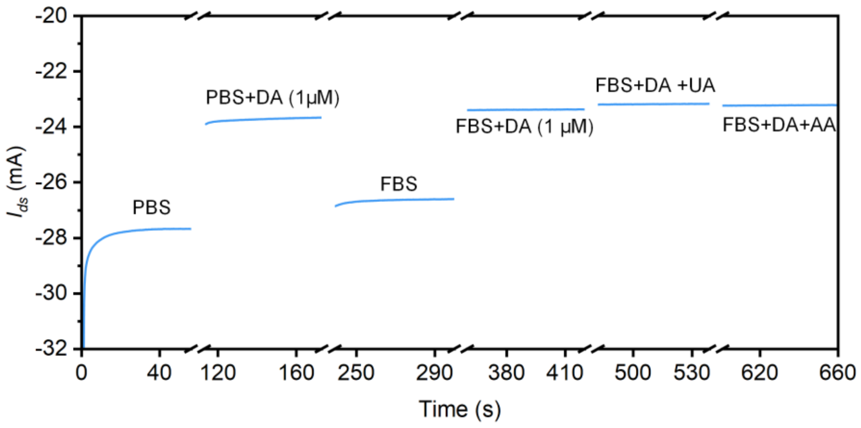

Figure S32. Real-time I_{ds} responses of MSDA-OECT in PBS and FBS with the DA and interfering substances. The concentration of the DA and interfering substances are 100 μM and 500 Mm, separately.

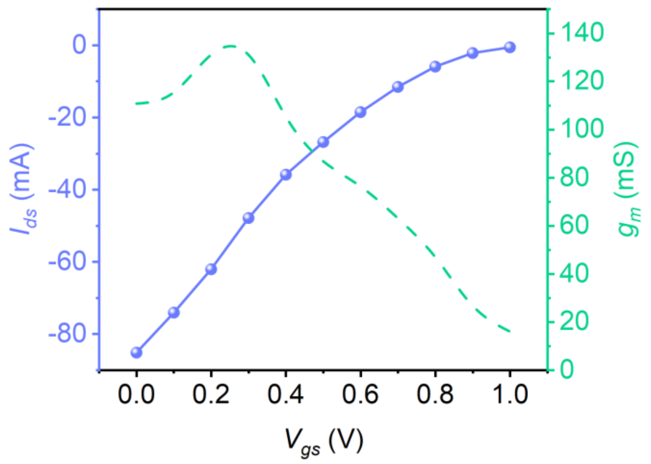

Figure S33. Transfer characteristics curve of the MSDA-OECT placed in the rabbit brain collected by the wearable sensing system (V_{ds} = -0.4 V).

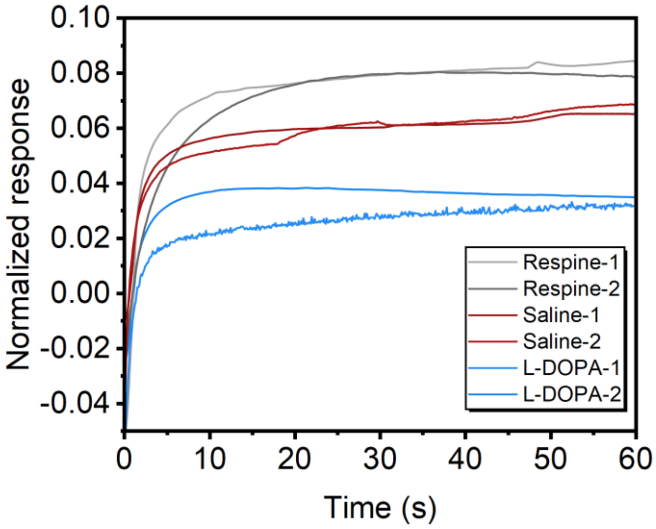

Figure S34. Real-time monitoring of I_{ds} of the MSDA-OECT placed in the rabbit brain. The I_{ds} of MSDA-OECT is collected every 5 minutes for 60s.

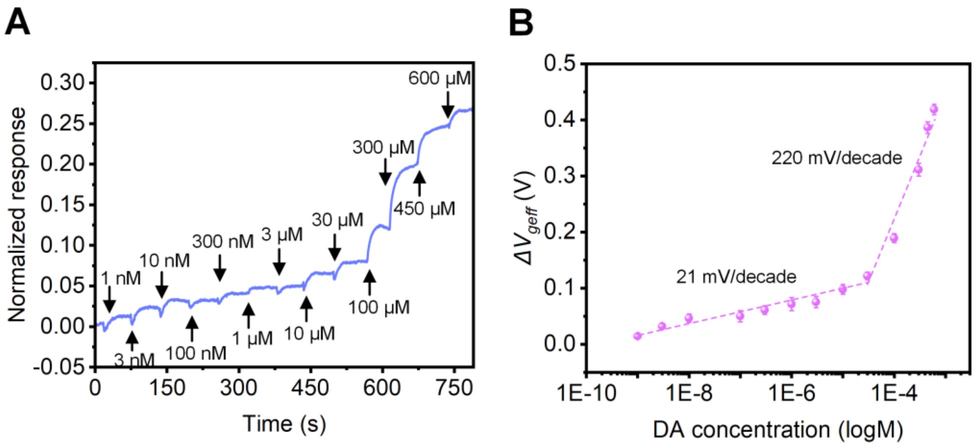

Figure S35. The I_{ds} responses and sensitivity of MSDA-OECT with 150 μm height RL-NC micropillars. (A) The I_{ds} responses of MS-DA OECT with 150 μm height RL-NC micropillars array to the successive addition of different concentration DA in PBS solution (V_{ds} = -0.4 V, V_{gs} = 0.2 V). (B) Relationship between V_{geff} and DA concentrations in logarithmic scale.

Table S1: Comparison of diffusion coefficient with varied electrodes.

Electrodes	Area (cm^2)	Peak current (A)	Diffusion coefficient (cm^2s^{-1})
Planar electrode	0.04	2.57×10^{-5}	2.29×10^{-6}
SD-NP micropillar electrode	0.144	1.49×10^{-4}	5.91×10^{-6}
RL-NC micropillar electrode	0.153	2.51×10^{-4}	3.29×10^{-5}

The D_0 is calculated using the Randles-Sevcik equation:

$$D^{1/2} = \frac{i_p}{269000 \times n^{3/2} A C v^{1/2}}$$

Where i_p is the redox peak current (A), n is the number of electrons in the redox (n=1, in this study), A is the electrode surface area (cm^2), C is the concentration of redox species (mol/cm^3), $v^{1/2}$ is the root mean square of scan rate (V/s).

Table S2: Comparison of the detection performance of DA sensors from the previously reported works and this work.

Sensor type	Gate/working electrode	Detection limit	Sensitivity	Flexibility	In vivo test	Ref.
Three-electrode	silver micropillars	0.5 fM	12.6 μA μM^{-1} (1 nM-10μM) 63.8μA μM^{-1} (10μM-1 Mm)	No	No	2
Three-electrode	COF[a]-modified carbon fiber	108 nM	10.76 nA μM^{-1} (25 nM-20μM)	No	Yes	3
OFET	carbon yarn with Nafion coating	3 nM	37 mV/decade	No	No	4
OFET	Au electrode	10 pM	-	Yes	Yes	5
OECT	graphene modified Pt electrode	5 nM	281 mV/decade (5 nM-1 μM)	No	No	6
OECT	CNT[b]/Pt NPs fiber	5 nM	-	Yes	Yes	7
OECT	Self-curled Au nanomembranes	3 μM	0.52 μM^{-1} (0-10 μM) 0.004 μM^{-1} (50-500μM)	Yes	No	8
OECT	Carbon fiber	5 nM	0.899 S M^{-1}	Yes	No	9
OECT	carbonized silk fabric	1 nM	60 mV/decade (0.1-30 μM)	Yes	No	10
OECT	N/O-codoped carbon cloths	1 nM	151 mV/decade (1-300 μM)	Yes	No	11
OECT	Multiscale micropillar	0.6 fM	4.2 mV/decade (1 fM-100 pM) 40 mV/decade (100 pM-100 μM) 254 mV/decade (100-600 μM)	Yes	Yes	This work

a. COF: covalent organic framework b. CNT: carbon nanotube

Movie S1: Fabrication of multiscale micropillars via temperature-induced self-assembly aerosol jet printing.

Movie S2: Fully printed fabrication of MSDA-OECT via aerosol jet printing.

Supplementary References

(1) Lavín, A.; de Vicente, J.; Holgado, M.; Laguna, M. F.; Casquel, R.; Santamaría, B.; Maigler, M. V.; Hernández, A. L.; Ramírez, Y. On the Determination of Uncertainty and Limit of Detection in Label-Free Biosensors. Sensors 2018, 18 (7).

(2) Ali, M. A.; Hu, C. S.; Yuan, B.; Jahan, S.; Saleh, M. S.; Guo, Z. T.; Gellman, A. J.; Panat, R. Breaking the Barrier to Biomolecule Limit-of-Detection via 3D Printed Multi-Length-Scale Graphene-Coated Electrodes. Nat. Commun. 2021, 12 (1), 7077.

(3) Zhou, L.; Yang, R. J.; Li, X. R.; Dong, N.; Zhu, B. Y.; Wang, J. J.; Lin, X. Y.; Su, B. COF-Coated Microelectrode for Space-Confined Electrochemical Sensing of Dopamine in Parkinson's Disease Model Mouse Brain. J. Am. Chem. Soc. 2023, 145 (43), 23727-23738.

(4) Xi, X.; Tang, W.; Wu, D. Q.; Shen, C. C.; Ji, W.; Li, J.; Su, Y. Z.; Guo, X. J.; Liu, R. L.; Yan, F. All-Carbon Solution-Gated Transistor with Low Operating Voltages for Highly Selective and Stable Dopamine Sensing. ACS Sens. 2023, 8 (3), 1211-1219.

(5) Wu, G. F.; Zhang, N. N.; Matarasso, A.; Heck, I.; Li, H. J.; Lu, W.; Phaup, J. G.; Schneider, M. J.; Wu, Y. X.; Weng, Z. Y.; et al. Implantable Aptamer-Graphene Microtransistors for Real-TimeMonitoring of Neurochemical Release in Vivo. Nano Lett. 2022, 22 (9), 3668-3677.

(6) Liao, C. Z.; Zhang, M.; Niu, L. Y.; Zheng, Z. J.; Yan, F. Organic Electrochemical Transistors with Graphene-Modified Gate Electrodes for Highly Sensitive and Selective Dopamine Sensors. J. Mat. Chem. B 2014, 2 (2), 191-200.

(7) Wu, X. Y.; Feng, J. Y.; Deng, J.; Cui, Z. C.; Wang, L. Y.; Xie, S. L.; Chen, C. R.; Tang, C. Q.; Han, Z. Q.; Yu, H. B.; et al. Fiber-Shaped Organic Electrochemical Transistors for Biochemical Detections with High Sensitivity and Stability. Sci. China-Chem. 2020, 63 (9), 1281-1288.

(8) Ferro, L. M. M.; Merces, L.; De Camargo, D. H. S.; Bufon, C. C. B. Ultrahigh-Gain Organic Electrochemical Transistor Chemosensors Based on Self-Curled Nanomembranes. Adv. Mater. 2021, 33 (29), 2101518.

(9) Li, W. Q.; Jin, J.; Xiong, T. Y.; Yu, P.; Mao, L. Q. Fast-Scanning Potential-Gated Organic Electrochemical Transistors for Highly Sensitive Sensing of Dopamine in Living Rat Brain. Angew. Chem.-Int. Edit. 2022, 61 (31), e202204134.

(10) Ji, W.; Wu, D. Q.; Tang, W.; Xi, X.; Su, Y. Z.; Guo, X. J.; Liu, R. L. Carbonized Silk Fabric-Based Flexible Organic Electrochemical Transistors for Highly Sensitive and Selective Dopamine Detection. Sens. Actuator B-Chem. 2020, 304, 127414.

(11) Xi, X.; Wu, D. Q.; Ji, W.; Zhang, S. N.; Tang, W.; Su, Y. Z.; Guo, X. J.; Liu, R. L. Manipulating the Sensitivity and Selectivity of OECT-Based Biosensors via the Surface Engineering of Carbon Cloth Gate Electrodes. Adv. Funct. Mater. 2020, 30 (4), 1905361.
